# Progress on pharmaceutical drugs, plant extracts and ionic liquids as corrosion inhibitors

**DOI:** 10.1016/j.heliyon.2019.e01143

**Published:** 2019-02-01

**Authors:** Lekan Taofeek Popoola

**Affiliations:** Unit Operation and Material Science Research Laboratory, Chemical and Petroleum Engineering Department, Afe Babalola University, Ekiti State, Nigeria

**Keywords:** Safety engineering, Organic chemistry, Industrial engineering, Bioengineering, Materials science, Chemical engineering

## Abstract

In the past, lives and wealth have been lost due to corrosion in almost all engineering fields. Not only this, the cost of reviving damaged equipments in the industry due to corrosion contributed a lot to the gross domestic product of a nation. Thus, all hands must be on desk to combat this harzadous act via time to time research on its final resolution. However, current research works have revealed effective and reliable corrosion inhibitors from pharmaceutical drugs, plant extracts and ionic liquids as organic green corrosion inhibitors (OGCIs) with accommodative attributes such as being environmentally friendly, readily available, biodegradable, non-harmful, relatively cheap and many others to mention a few. This paper opens readers mind into the detailed classifications, mechanisms and active functional groups of these eco-friendly OGCIs. Not only the corrosion efficiency calculation ways but also influencing factors on efficiency were presented. Plant extracts, pharmaceutical drugs, ionic liquids and synthetic inhibitors, as among major sources of OGCIs, used in preventing material corrosion in corrosive media were separately and comprehensively examined. The significance of values obtained from simulating presented mathematical models governing OGCIs kinetics, adsorption isotherm and adsorption thermodynamics was also included. In conclusion, beneficial recommendations for both current and prospective researchers in the field of Corrosion Engineering were presented.

## 1 Introduction

**Fig. 1:**
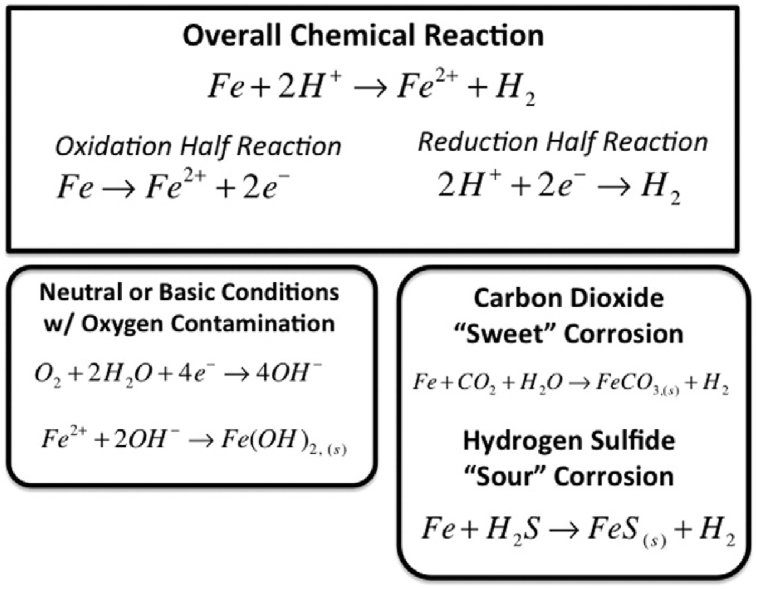
Chemical reactions of corrosion process [16].

Metal degradation due to its contact with aqueous corrosive surroundings (air, moisture or soil) [1] through direct chemical or electrochemical reaction to form noble compounds [2] results to a phenomenon called corrosion. It is an interfacial material (polymer, metal, concrete, wood and ceramic) reaction (irreversible) with its environment which results in material consumption or in dissolution into the material of an environmental component [3] according to IUPAC. Corrosion is an environmental threat with economic, conservation and safety impacts in various engineering applications such as building construction, chemical, automobile, mechatronics, metallurgical, medical and so on [4]. Various forms of material corrosion under different environments had been discussed [5]. A summary of common corrosion types and their respective mechanisms have been presented in Table 1. The impact of corrosion cost caused by both direct and indirect damage of materials on the economic status of the world is becoming alarming. Research works conducted from 1999 to 2001 on corrosion costs and preventive strategies in both United States and United Kingdom revealed 3.1% of their Gross Domestic Product (GDP) as cost spent only on direct corrosion damage [6]. The economics of corrosion can be grouped into capital costs (equipment replacement, redundant equipment and excess capacity), control costs (maintenance, repair and corrosion control), design costs (materials of construction, special processing and corrosion allowance) and associated costs (technical support, product loss, insurance and equipment inventory). However, studies have shown that corrosion cost can be reduced by 15–20% if low cost novel corrosion control techniques are applied [5]. Thus, there is need to develop novel techniques and methods to tackle this dangerous phenomenon from existing prominent ones which are protective coatings and linings, cathodic/anodic protection and corrosion inhibitors. Table 2 gives a summary of ways of controlling corrosion. However, results of numerous researches conducted in anticorrosion materials applications in previously mentioned engineering fields revealed using corrosion inhibitors as the most effective and simple approach of preventing deleterious degradation of metals and alloys in corrosive media [7, 8]. Fig. 1 depicts summary of chemical reactions of corrosion process.

**Table 1:** Common corrosion types [9, 10, 11, 12].

Corrosion type	Mechanism
Pitting	This is localized corrosion attack due to neutralization salts presence on metal surface causing some parts to corrode quickly (acting as anode) but some are free from corrosion (acting as cathode). Thereby, causing deep holes.
Galvanic	Flow of electrons between two dissimilar metals resulting from potential difference existence between them when subjected to corrosive media thereby causing corrosion. The less resistant metal acted as anode while the most resistant acted as cathode.
Uniform	Uniform occurrence of corrosion on all areas of metal at the same rate.
Crevice	Occurrence of corrosive liquid capture in between metal gaps resulted into concentration cell corrosion.
Erosion	Exposure of metal surface to a high velocity corrosive fluid thereby, exposing the stripped surface to more corrosion attack.
Stress corrosion cracking	Mechanical tensile stress and hostile chemical corrosive medium caused formation of fracture in metal structure thereby exposing the fractured surface or point to more corrosion attack.
Intergranular	Corrosion occurrence on metal grain boundaries.
Corrosion fatigue	Corrosion due to combined effects of cyclic stress and corrosive medium.
Fretting	Advanced erosion-corrosion due to metal fretting and corrosive medium combined effects.

**Table 2:** Corrosion control ways [13, 14, 15].

Control method	Description
Material selection	The sequential steps required in picking appropriate material include: preliminary selection, laboratory testing, laboratory result interpretation, economic analysis of apparently suitable materials and final selection. The finally selected material should have high mechanical strength, high corrosion resistance and low cost.
Surface coating	This involves the use of anticorrosive protective coating to form a physical barrier between corrosive environments and material. It can be sub-divided into metallic (a more noble layer of other metal used to coat the material) and non-metallic (organic coatings such as paints, lacquers and coal tar; and inorganic coatings such as porcelain enamels, chemical-setting silicate cement linings, glass coatings and linings are being used to isolate the material from corrosive environment).
Excellent Equipment Design (EED)	EED enables application of novel design principles which put cost reduction, time and future corrosion maintenance and repair into consideration. Typical examples of how EED can minimize corrosion include: avoid dissimilar metal contact when electrolyte is present, avoid crevice corrosion by joining different sections using welding rather than riveting, double section of the material under extreme degree of turbulence flow regime to avoid erosion-corrosion, equipment vibration should be avoided, storage tanks should be designed for easy drainage and so on.
Electrical protection	This could be classified as either cathodic protection (minimizes metal surface corrosion by making it the cathode of an electrochemical cell such that potential difference between anode and cathode is minimized simultaneously) or anodic protection (which is based on the principle of passivity executed by connecting material to be protected to an external d.c power supply positive pole).
Corrosion inhibitors	These are substances added in small concentrations/amount to a corrosive environment to reduce or stop electrochemical corrosion reactions occurring on a metal surface. They could be organic or inorganic based on their sources and areas of application.

**Fig. 2:**
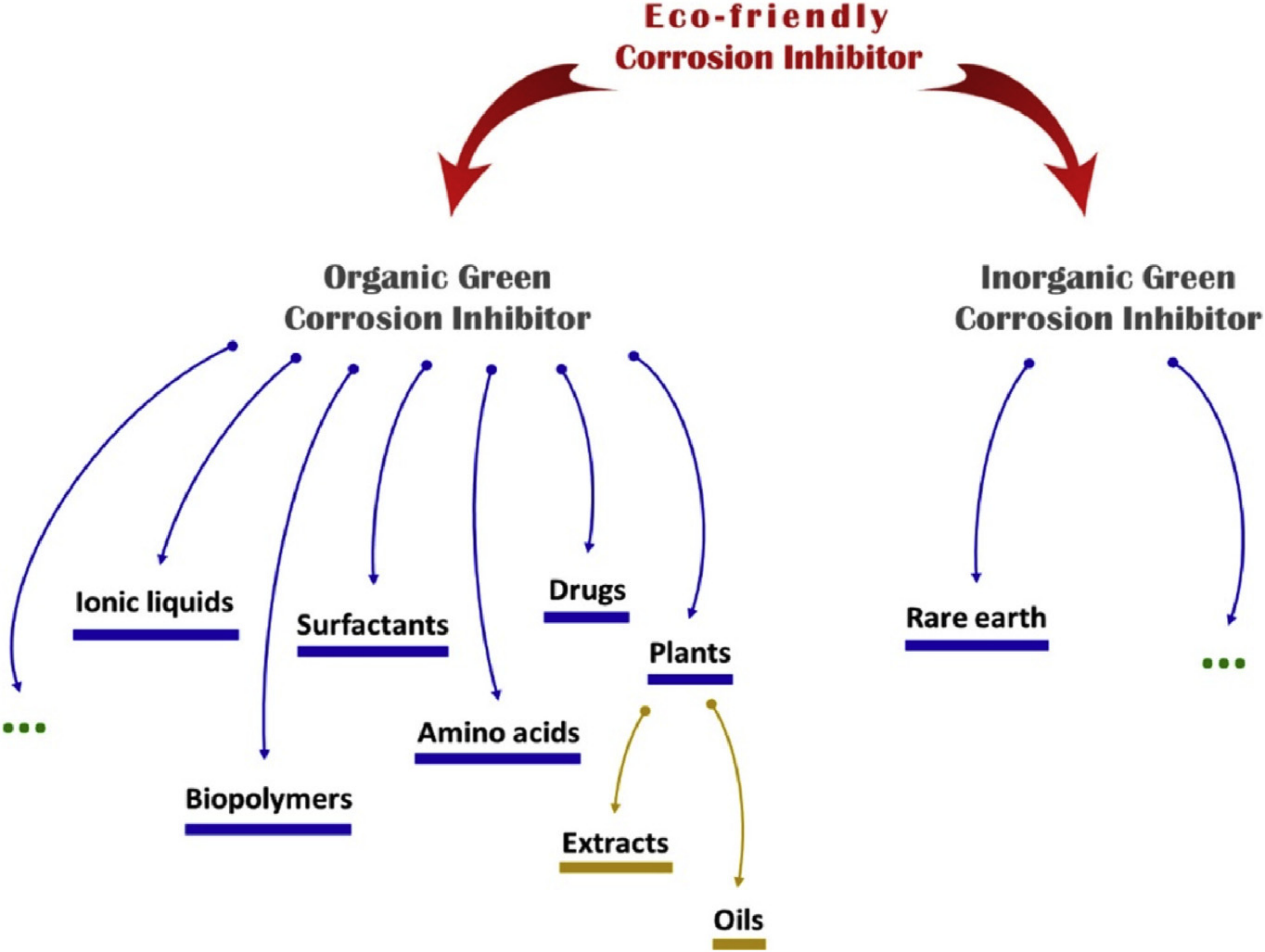
Sources of eco-friendly OGCIs [23].

Corrosion inhibitors (CIs) minimize or avert corrosion when added in small concentrations to a corrosive medium [17] by forming monomolecular film-adsorbed surface [18] which obstructs direct contact between metal and corrosive agents [19]. They have been classified based on sources (as organic or inorganic) and techniques (synthesized or extracted). Thus, there is required to look for not only applicable corrosion inhibitors but also those that are economically viable and environmentally friendly. However, synthetic organic corrosion inhibitors (SOCIs) and traditional inorganic corrosion inhibitors (TICIs) such as chromates and lead have been known to have restrictive environmental regulations [20] due to their hazardous effects. Many of the SOCIs are not biodegradable and get accumulated in the environment constituting nuisance to human health or ecological systems [21] whose removal is complicated and expensive [22]. These environmental issues have called for replacement of these TICIs and SOCIs with natural organic compounds sourced from spices, naturally existing aromatic herbs and medicinal plants that can hinder corrosion of materials in corrosive media called organic green corrosion inhibitors (OGCIs) which are inexpensive, harmless, readily obtainable and environmentally accomodative. Fig. 2 presents various sources of eco-friendly OGCIs.

Historically, using OGCIs started in early 1930s when extracts from plant such as *chelidonium majus* (celandine) was first utilized for H_2_SO_4_ pickling baths [24]. After then, researchers around the world found interest in using green anticorrosive agents extracted from several natural plants [25]. Seeds, fruits, leave and flowers of natural plants such as *Justicia gendarussa* plant extract [26], khillar [27], olive leaves [28], *Phyllantus amaratus* [29] and *Murraya koenigii* leaves [30] were extracted and applied as corrosion inhibitors. Results revealed natural plants extracts to be easily obtainable, biodegradable and harmless [31] with remarkable potential of inhibiting corrosion reaction.

### 1.1 Mechanisms of OGCIs

Corrosion inhibition efficiency of OGCIs has been linked to the availability of organic compounds having nitrogen, oxygen, phosphorus and sulphur atom [32] which have shielding effect and corrosion-inhibiting potentials for materials attack. Their increasing order of corrosion inhibition efficiency has been stated to be oxygen < nitrogen < sulphur < phosphorus [33]. OGCIs exhibit their inhibition action via physi- or chemisorption onto metal/solution interface by removing molecules of water on the surface for compact barrier film formation [34]. Occurrence of coordinate covalent bond by interaction between lone pair and π-electrons available in the molecules of OGCIs with the vacant metal *d*-orbitals is also experienced [35]. Nevertheless, compounds adsorption on the metal surface is enhanced by *p–d* bonds formation as a result of *p-*electrons overlap to the *3d* vacant orbital of iron atom [36] due to the availability of N, O, S atoms and organic structures double bonds [37].

Generally, adsorption types can be distinguished by the occurring mechanisms which could be physisorption, chemisorption, interaction between metal and *p*-electrons or mixture of the aforementioned [38]. The chemical structures of OGCIs, inhibitor molecule charge distribution and metal surface charge determine the process of adsorption. In physisorption, electrostatic attractive force binds ionic charges on OGCIs molecules with electric charged metal surface while chemisorption occurs via sharing of free electron pairs or transfer of charge to produce strong chemical bonds between non-ionic OGCIs molecules and metal [39]. However, the chemisorption bond strength is a function of functional group electron density present on the donor atom and group polarizability. OGCIs inhibition efficiency is improved when one of H atoms attached to the C in the heterocyclic ring is displaced by any of –CHO, –NO, –COOH or -NH_2_ substituent group [40]. Also, cathodic or anodic reactions are being retarded when metal electron density changes at the point of attachment. Consumption of electrons at the cathode occurred while they are furnished at the anode [41].

### 1.2 Classifications of OGCIs

Corrosion inhibitors from greeners can either be scavengers or interface inhibitors. Scavengers reduce medium corrosivity via aggressive substances scavenging while interface inhibitors inhibit corrosion through film formation at the environment/metal interface. The scavengers work in alkaline and almost neutral solutions by cathodic-oxygen reduction reaction simply written as Eq. (1). The interface inhibitors are classified as either vapour-phase or liquid-phase. The vapour-phase inhibitors provide temporal atmospheric corrosion protection especially in closed environments by loosely impregnating wrapping paper inside a closed container [42]. The OGCIs transport and metal surface interaction occur during vapor-phase inhibition process.(1)O_2_ + 2H_2_O + 4*e*^*−*^ 4OH^−^

However, the most prominent are the liquid-phase inhibitors which are further sub-divided into cathodic, anodic or mixed OGCIs based on the reaction type inhibit which could be any of cathodic, anodic or both electrochemical reactions. In anodic OGCIs, hydroxides, oxides or salts are produced to enhance passivating films formation which inhibits anodic metal dissolution reaction. Their mechanism is best explained by an active-passive metal polarization diagram. In cathodic OGCIs, corrosion is controlled either by cathodic poisoning or cathodic precipitation. In the former, sulfides and selenides which act as cathodic poisons are adsorbed on the surface of the metal to form protective films that reduce rate of cathodic reaction through oxygen diffusion minimization on metal surface. In the latter, insoluble compounds such as carbonates of calcium and magnesium are precipitated on metal surface in order to increase alkalinity at cathodic sites. Generally, hydrogen ions reduction to hydrogen atoms to form molecules of hydrogen as written in Eqs. (2) and (3) occurs in acidic solution while cathodic reaction occurs via reduction of oxygen in alkaline solution.(2)H^+^ + *e*^*−*^ H(3)2H  H_2_

Lastly, approximately 80% of OGCIs are categorised as mixed inhibitors which protect metal from corrosion by chemisorption, physisorption and film formation. Physisorption is facilitated by electrostatic attraction of negatively charged (anionic) OGCI with positively charged metal surface. Chemisorption process is slower than physisorption such that inhibition rate and adsorption increase as temperature increases [43]. Corrosion protection also increases with increase in polymeric films produced as a result of OGCI molecules adsorbed which are subjected to reactions on metal surface. Insoluble adherent films that avert solution access to the metal provide effective inhibition.

### 1.3 Active functional groups in OGCIs

The OGCIs active ingredients consist of phytochemical constituents known to be functional groups with N, O, S, P or Se hetero atoms via which they are attached onto the metal surface [44, 45]. Compounds of OGCIs having abundant *p*-electron and functional electronegative groups with conjugated double or triple bonds have been shown to be most effective [46]. The inhibitor molecule efficiency to cover enough surface area is increased due to the attached groups to the parent chain. In lieu of this, bonding strength of the group on the metal is enhanced by the presence of peculiar repeating units (methyl and phenyl groups) of the parent chain and additional substituent groups. As the substituents number on the functional group of an inhibitor increases, the inhibitive energy in general also increases. Studies have shown that OGCIs molecules with –OH and –OCH_3_ electron releasing substituents proved to have better efficiency than parent molecule having no substituents [47]. Also, heterocyclic compounds have exhibited higher corrosion inhibition efficiency as they easily on metallic surface via their π- and non-bonding electrons, aromatic rings and polar functional groups which act as adsorption centers [36]. Table 3 presents some anchoring functional groups present in OGCIs.

**Table 3:** Some attaching functional groups in OGCIs [48].

Fuctional Group	Name	Fuctional Group	Name
-OH	Hydroxy	-NH_2_	Amino
-C-N-C-	Amine	-SH	Thiol
-NO_2_	Nitro	-C≡C-	-yne
-CONH_2_	Amide	-S=O	Sulfoxide
-COOH	Carboxy	-NH	Imino
-S-	Sulfide	-N=N-N-	Triazole
-C=S-	Thio	-C-O-C-	Epoxy
-P=O	Phosphonium	-P-	Phospho
-Se-	Seleno	-As-	Arsano

Some prominent compounds such as benzoic acid [49], benzotriazole [50], thiourea [48], flavonoids [51], carbohydrates [52], tannins [53] and tryptamine [54] containing these active functional groups whose sources are from natural plants had been applied as corrosion inhibitors for many metals. Flavin mononucleotide from grape pomace extracts was detected as a good OGCI for hot rolled steel in acidic medium [51]. Its corrosion inhibition potential lies in the presence of heterocyclic isoalloxazine ring anchored to sugar alcohol-ribitol obtained from *D* (-) pentose sugar (ribose) which consists of a phosphate monosodium salt and three antisymmetric carbons. The bark of Rhizophora Racemosa stem investigated to be very rich in tannins has been stated as the most effective OGCI for mild steel. Its basic structure contains residues of garlic acid attached to glucose through bonds of glycosidic [53] with arrays of hydroxyl and carboxyl groups enhancing molecules adsorption on corroding mild steel surfaces. *Chamaerops humilis* plant extract which is also rich in tannins is effective in inhibiting corrosion of mild steel in 0.5M sulfuric acid solution with 5% ethanol additive [55]. Tryptamine, a derivative of the tryptophan, proved effective in inhibiting ARMCO iron corrosion in deaerated 0.5M sulphuric acid within temperature range of 25–55^∘^C. Table 4 presents various sources of OGCIs with their respective functional groups and inhibitory roles while Table 5 contains chemical structures of OGCIs and their areas of application.

**Table 4:** Sources of OGCIs, functional groups and corrosion inhibitory roles.

OGCI source	Functional groups and compounds	Corrosion inhibitory roles	Reference
*Ginkgo biloba* leave extracts	Flavonoids and terpenoids; phenol groups and aromatic rings.	Terpenoids: Quercetin adsorption on mild steel surface based on interactions of donor–acceptor between *O* and aromatic ring *p*-electrons and surface iron vacant *d* orbitals. Flavonoids: Oxygen-adsorption corrosion inhibited via its oxidation to benzoquinone by O_2_ resolved in the solution.	[56]
*Rothmannia longiflora* extract	Monomethyl fumarate (MMF), 4-oxonicotinamide-1-(1-β-D-ribofuranoside) (RBF) and D-mannitol (DMT)	-	[57]
*Petersianthus macrocarpus* plant	Petersaponin, β-sitosterol, and ellagic acid	Molecules adsorbed on surface of mild steel surface as a result of hydroxyl group and aromatic rings protonation. Constituent molecules have aromatic rings (π-electrons) with attached electron releasing groups. Also, increase of the ability of π-electrons to be bonded to vacant *d-*orbital in Fe.	[57]
Extract of *Ficus asperifolia*	Saponins, alkaloids, tannins anthraquinones, flavonoids and reducing sugars, n-hexane, ethylacetate, butanol	Electron donating ability was facilitated as a result of rich bond or hetero atoms present in the chemical structures. Thus, formation of complexes on material surface to inhibit corrosion was enhanced.	[58]
*Diospyros* Kaki L.f husk extracts	Vitamins, p-coumaric acid, gallic acid, catechin, flavonoids, carotenoids and condensed tannin	-	[59]
Gum arabic	Arabinogalactan, oligosaccharides, polysaccharides and glucoproteins	-	[60]
Tobacco extract	Polyphenols, terpenes, alkaloids, alcohols, carboxylic acids and nitrogen-containing compounds.	Corrosion inhibition on metals by electrochemical active due to fused benzene ring system with charge dislocation property.	[61]
Extract of green wild jute tree (*Grewa venusta*)	Polysaccharides, polyphenols (catechins and flavonoids) vitamins, tannins, minerals, volatile oils and alkaloids.	Mixed inhibitor corrosion inhibition action.	[54]
*Anthocleista djalonesis*	Iridoid glucoside (djalonenoside), Dibenzo-α-pyrone (djalonensone), ursolic acid, 3-oxo-Δ-4,5-sitosterone.	-	[62]
Guar gum	Polysaccharide mainly sugars galactose and mannose	1,4-linked mannose residues linear chain forming short side branches which later formed complexes on metal surface to inhibit corrosion.	[63]
Jatropha Curcas leave extract	Tannis, flavonids, terpenes, anthra-quinone, apigenin, cardiac glycoside, alkaloids, deoxy sugar, saponins, alpha-D-glucoside, sterols, stigmasterol and vitexin	Corrosion inhibition via formation of continuous complex metal ions on metal surface by polar groups.	[64, 65]
Extracts of banana peel	bananadine (3Z,7Z,10Z)-1-oxa-6-azacyclododeca-3,7,10-triene	-	[66]
Aloe vera plant extract	polysaccharides, glycoproteins, vitamins, mineral, and enzymes	-	[67]
Azadirachta indica	azadirachtin, salannin, meliantriol and nimbin	Inhibition effects due to electronic, geometrical coupled with binding property bases on the surface of metal.	[68]
Locust bean gum	galactomannan-type polysaccharides	-	[69]
Oil palm frond	phenolic constituents (*p*-hydroxybenzoic acid, syringic acid, vanillic acid, vanillin, *p*-hydroxybenzaldehyde, *p*-hydroxyacetophenone and syringaldehyde)	Lignin is cleaved to form aromatic carbonyl compounds (syringaldehyde and vanillin) via alkaline nitrobenzene oxidation to inhibit corrosion.	[70]
*Justicia gendarussa* plant extract	Friedelin, β-sitosterol, *o*-substituted aromatic amines lupenol, phenolic dimmers and flavonoids.	Corrosion inhibition of metal as a result of mixed type inhibitor actions.	[26]
Leaves and flowers extracts of *Heliconia rostrata*	Alkaloids, flavonoids, tannins, cellulose and polycyclic compounds.	Presence of heterocyclic constituents enhanced film formation over metal surface thus affording corrosion inhibition.	
Celery (Apium graveolens) seeds	Flavonoids, linoleic acid, d-limonene, sesquiterpene alcohols, coumarins, selinene, sedanolide and sedanonic anhydride.	-	[71]
Henna extract (*Lawsonia inermis*)	Lawsone, α-D-glucose, gallic acid and tannic acid	Mixed type corrosion inhibition mechanism with constituents order of inhibition efficiency of tannic acid ˂ α-D-glucose < gallic acid < henna extract < lawsone.	[72]

**Table 5:** Chemical Structures of OGCIs and their Areas of Application [13].

OGCIs general name	Structure	Corrosion inhibition application
Alkylamines (n = 2–12)		Primary amines and diamines active for corrosion inhibition in acidic media.
Diamines (n = 2–8)		
Cycloalkylic		
Aromatic		
Benzilamines		Secondary amines which inhibit corrosion of carbon steel in acidic media.
Etoxilated amines		
Alkyloximes		Oximes for carbon steel corrosion inhibition in acidic media.
Aromatics		
Alkylnitriles		Nitriles good for corrosion inhibition of carbon steel in acidic media.
Aromatics		
Ureas y thioureas		Excellent for copper alloys and carbon steel corrosion inhibition in acidic media.
Amides		Amides y thioamides excellent for carbon steel corrosion inhibition in acidic media.
Thioamides		
Imidazoles		Active for copper alloys and carbon steel corrosion inhibition in basic media.
Benzoazoles		Good for copper alloys and carbon steel corrosion inhibition in basic media.
Imidazolines		Excellent for corrosion inhibition of carbon steel in acidic media.
Pyridines		Excellent for corrosion inhibition of carbon steel in acidic media.
Triazoles		Copper alloys corrosion inhibitors in basic media.
Benzotriazoles		Copper alloys corrosion inhibitors in basic media.
Tetrazoles		Good for copper alloys corrosion inhibition in basic media.
Polyvinyls		Excellent for corrosion inhibition of carbon steel in acidic media.
Polyesters		Good for carbon steel corrosion inhibition in acidic media.

### 1.4 Factors influencing OGCIs efficiency

OGCIs' efficiency in inhibiting corrosion is a function of their adsorption characteristics on the metal surface. Factors that have been considered by previous studies affecting OGCIs inhibition efficiency depend majorly on their structure, concentration, temperature and exposure time. Increase in OGCIs concentration results in simultaneous decrease in corrosion rate with increase in inhibition efficiency which approaches optimum level at certain concentration value. This resulted from formation of additional inhibitor molecules being adsorbed on the surface of metal which makes it complex for further corrosive attack to occur by the electrolyte solution. The dissolution of metal increases with corrosion exposure period in the presence of OGCIs. This is linked to previously adsorbed inhibitor molecules from metal surface resulting from partial desorption. Corrosion rate increases linearly as temperature increases such that equilibrium exists between adsorption and OGCIs molecules desorption at the surface of metal at a particular temperature. Increase in temperature as a result of higher desorption rate makes the equilibrium to shift until its' re-establishment at various equilibrium constant values. Thus, OGCIs inhibitive protectiveness decreases with increasing temperature. As earlier said, OGCIs' structural behaviour has a great influence on their efficiencies in corrosive media. Presence of heteroatom in OGCIs molecule enhanced their adsorption onto metal surface through the formation of adsorptive bond by Lewis acid-base reaction in which OGCIs and metal act as electron donor and acceptor respectively. The strength of adsorption bond is a function of electron density and polarizability of reaction centre. Conclusively, studies have shown surface active OGCIs adsorption to increase with increasing molecular weight and dipole moment.

### 1.5 Measuring OGCIs efficiency

The first step required in measuring efficiency of OGCIs is the preparation of metal sample to be examined for corrosion. The selection of metal coupons for checking OGCIs efficiency is vital as small changes in metal composition or available impurities during fabrication reflect in the results obtained [73]. The metal composition should as much as possible be relevant to metals relating to corrosion problem. Of all the methodologies for measuring OGCIs efficiency available in literatures, weight loss, electrochemical impedance spectroscopy, linear polarization resistance and potentiodynamic polarization are prominent ones.

#### 1.5.1 Weight loss measurement (WLM)

Many researchers used test solutions prepared from actual field solution for corrosion testing while some used synthetic solutions prepared from analytical grade reagents in the absence of the former for WLM. Before the OGCIs efficiency is determined using WLM, metal samples are cleaned by polishing with abrasive paper of different grades and washed thoroughly using solvents (acetone, ethanol and distilled water) after which they are dried at room temperature. Vernier caliper is used to measure the dimensions of the metal specimen. The prepared metal coupons are then weighed prior to immersion using high-accuracy digital balance. The thoroughly rinsed corroded metal coupons are re-weighed after specified period of exposure time to check the weight loss. The corrosion rate [72], surface coverage degree *θ* [74] and percent corrosion inhibition efficiency η% [75] can then be evaluated using Eqs. (4), (5), and (6) respectively. The influence of the OGCI in preventing corrosion attack of the metal coupons is checked by samples weight loss measurement in the absence and presence of OGCI. WLM technique is simple and reliable as it forms the basic fundamental method of measuring OGCIs efficiency in many corrosion-monitoring programmers.

(4) $$CR=\frac{w_{1}-w_{2}}{At}$$

(5) $$\theta =\frac{w_{1}-w_{2}}{w_{1}}$$

(6) $$\eta \%=\frac{w_{1}-w_{2}}{w_{1}}\times 100\%$$

where CR = rate of corrosion ($g\;cm^{-2}\;{\text{hr}}^{-1}$), $w_{1}$ = metal coupons weight loss in the absence of OGCI (grams), $w_{2}$ = metal coupons weight loss in the presence of OGCI (grams), A = metal coupon surface area (cm^2^), t = immersion time (hours).

However, there are cases where the inhibition efficiency of OGCI is enhanced as a result of combination with another OGCI such that the inhibition efficiency is increased by an appreciable value. This is called synergism effect which can be quantified using Eq. (7) [76].

(7) $$S=\frac{1-\theta_{A}-\theta_{B}+\theta_{A}\theta_{B}}{1-\theta_{AB}}$$

where *θ*_*A*_ and *θ*_*B*_ = respective surface area coverage by compound A and B when acting separately; and *θ*_*AB*_ = surface area coverage obtained for mixture of A and B. When the value of *S* approaches 1, interaction between A and B is negligible. If S is greater than 1, it reveals existence of synergism while a value of S being less than 1 signifies opposite effect between A and B [77].

#### 1.5.2 Potentiodynamic polarization (PDP)

**Fig. 3:**
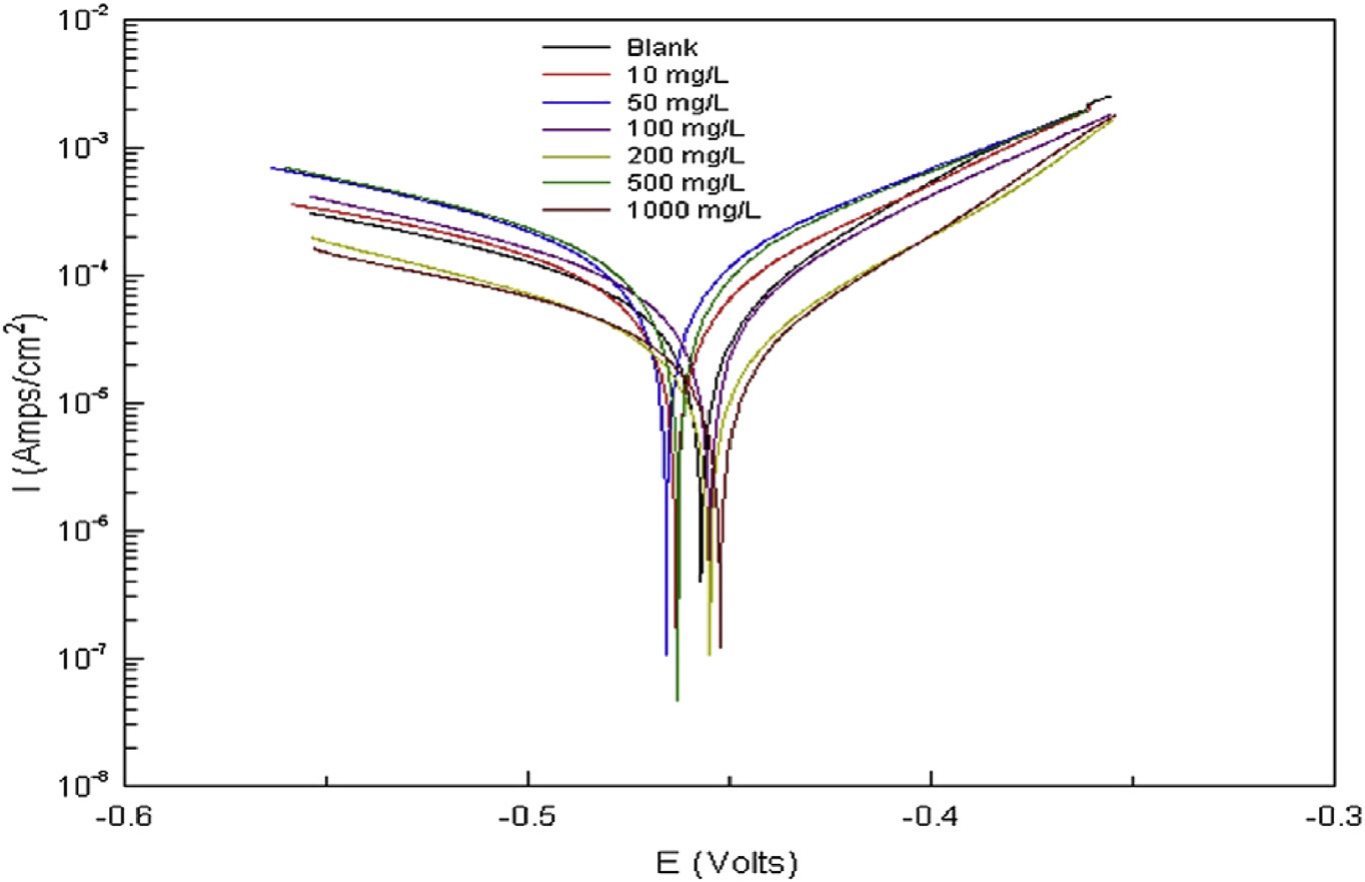
Polarization curves for Q235A steel corrosion in 1M HCl in the absence and presence of varying concentrations of persimmon husk extracts as OGCI [59].

Potentiodynamic polarization is another means of measuring OGCIs efficiency, corrosion rate and corrosion mechanism protection through electrochemical-based measurements. In most cases, the basic lab set up involves using three electrodes in the electrochemical cell which are working, counter and reference electrodes for the measurement immersed in the test solution of known volume and concentration. Platinum electrode [78] and graphite rod [79] are mostly used as the counter electrode while saturated calomel electrode [57] and Ag/AgCl aqueous electrode as the reference electrode. The working electrode is the metal substrate under examination. The voltage *(V)* of the system is measured and controlled by the reference electrode while the current *(I)* is measured by the counter electrode. As the electrochemical reactions progress, open circuit potential (*E*_*ocp*_) of the metal fluntuates. At equilibrium, a stable value is then measured afterwhich potentiodynamic polarization scan is performed. Application of a potential from a value below the initially measured *E*_*ocp*_ to higher potential (between -0.25 to +0.25 V) then gives the Tafel plot. The corrosion current (*i*_*corr*_) and corrosion potential (*E*_*corr*_) can then be measured from the plots. Fig. 3 represents typical polarization curves for Q235A steel corrosion in 1M HCl in the absence and presence of varying concentrations of persimmon husk extracts as OGCI. Corrosion rate is measured using Eq. (8) [80] while corrosion inhibition efficiency (η%) is calculated by measuring *i*_*corr*_ in the presence and absence of OGCIs using Eq. (9) [81].

(8) $$CR\;=\;\frac{i_{corr}\times K_{1}\times EW}{\rho }$$

(9) $$\eta \%\;=\;\frac{i_{corr}^{o}-i_{corr}^{1}}{i_{corr}^{o}}\times 100\%$$

where *CR =* corrosion rate (mm/yr), *K*_*1*_ = conversion factor = 3.27 × 10^−3^ mm g/μA cm yr, *EW* = equivalent weight (grams), ρ = density (grams/cm^3^), *A* = sample area (cm^2^), $i_{corr}^{o}$ and $i_{corr}^{1}$= corrosion current densities values (μA/cm^2^) in the absence and presence of OGCIs molecules respectively.

#### 1.5.3 Electrochemical impedance spectroscopy (EIS)

**Fig. 4:**
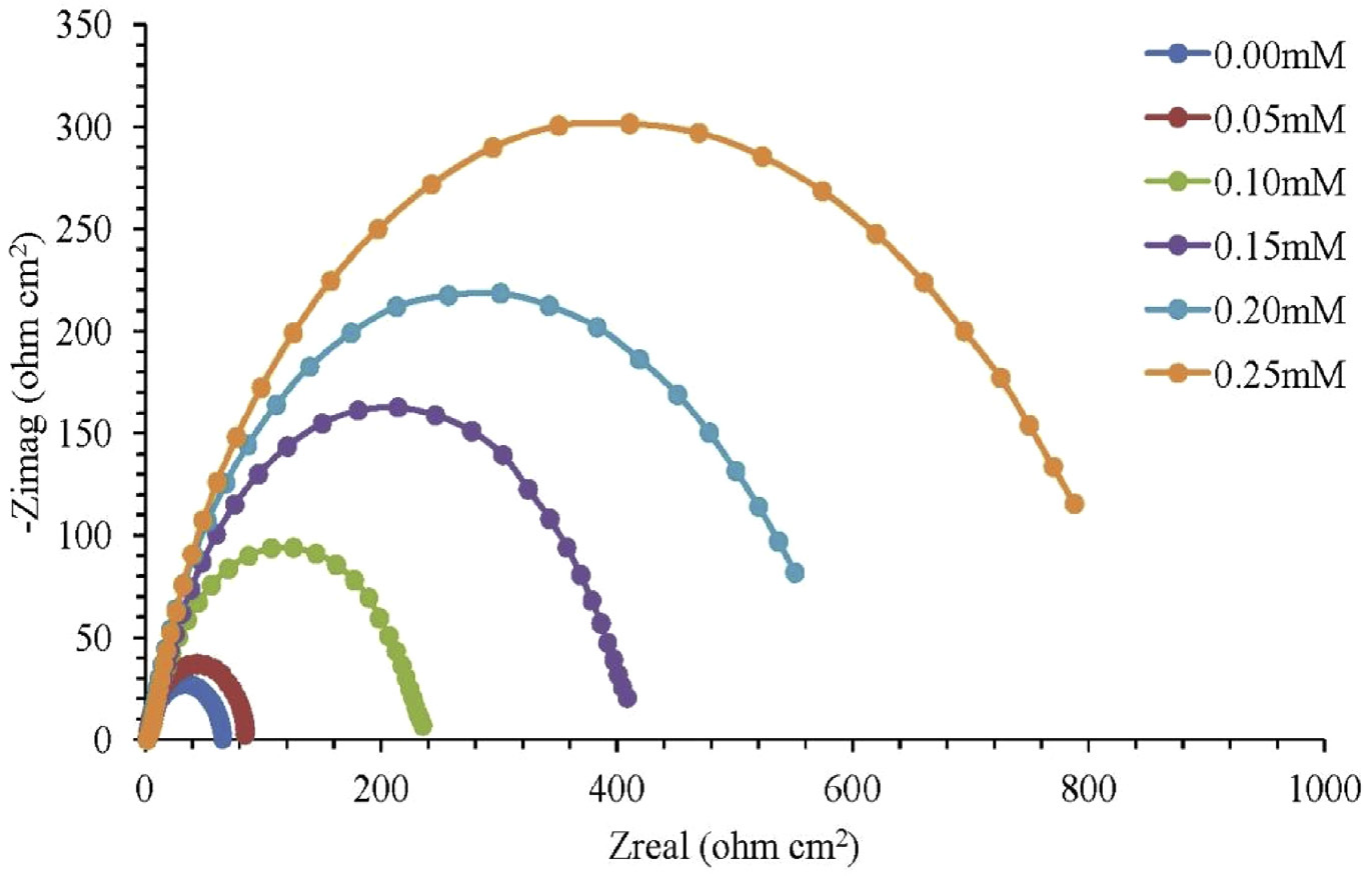
Mild steel Nyquist plot in 1M H_2_SO_4_ at 30 °C for varying OGCI concentrations [86].

**Fig. 5:**
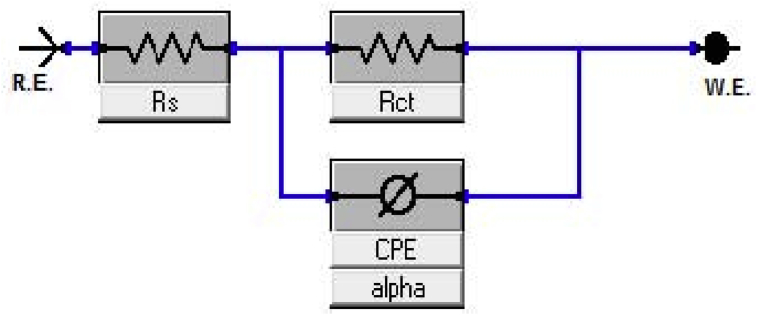
Equivalent circuit for fitting EIS data showing the positions of CPE, R_ct_ and R_s_[87].

EIS is an essential method of monitoring *in situ* electrochemical changes with critical understanding of physical processes occuring at the metal/electrolyte interface [82] such that information relating to electrode kinetics, surface properties and mechanistic could be gotten from impedance diagrams [83]. Just like PDP, the experiment is conducted in a three-electrode electrochemical cell with small potential upsetting between 5-50 mV of AC voltage over frequencies variation between 100 kHz and 10 mHz [84]. The EIS parameters are obtained using experimental EIS spectral (Nyquist plot) obtained with the aid of suitable circuits from values of frequencies which correspond to real (Z′) and imaginary (Z″) impedance values. A typical Nyquist plot for examining mild steel in 1M H_2_SO_4_ at 30 °C by means of new schiff base extract with different concentrations as OGCI is shown in Fig. 4. The adopted equivalent circuit (shown as Fig. 5) comprises *R*_s_ (electrolyte solution resistance), in series with parallel arrangement of constant phase element (CPE) and *R*_ct_ (charge transfer resistance) [85] modelled in a system of metal substrate, adsorbed inhibitors and electrolyte solution.

However, a study had used polarization resistance (*R*_*p*_) obtained as real impedance difference at reduced and higher frequencies to replace the usual charge transfer resistance (*R*_*ct*_) [87]. The polarization resistance (*R*_*p*_) was noticed to include *R*_*ct*_, accumulation resistance (*R*_*a*_) resulting from species accumulated at metal/electrolyte interface, diffusion layer resistance (*R*_*d*_) and inhibitor film resistance (*R*_*f*_) at metal surface. Anode-cathode charge transfer causes metal oxidation which is usually obstructed by the presence of solvent molecules in aqueous acid solution. The resistance by the electrolyte solution is called the solution resistance (*R*_s_). The charge transfer resistance (*R*_ct_) represents protective film capacity of adsorbed organic molecules on metallic surface to impede charges transfer the metal/solution interface. Impedance parameters which include *R*_*p*_*, n, C*_*dl*_ and *η%* could then be obtained from Nyquist plot by the equivalent circuit.

For better explanation of a frequency independent phase shift existing between an applied alternating potential and its current response, a constant phase element (CPE) being represented mathematically as Eq. (10) is used instead of capacitance (*C*) [26].

(10) $$Z_{CPE}=\frac{1}{A}{\left(j\omega \right)}^{-n}$$

where $Z_{CPE}$ = CPE impedance, *A* = CPE constant, ω = angular frequency, *j* = imaginary number (i.e *i*^2^ = −1), *n* = phase shift exponent which is a measure of surface irregularity/inhomogeneity. The significance of *n* is that a lower surface roughness is obtained at higher value of *n* and vice versa. Also, *n* value determines the nature of constant phase element and states what *A* in Eq. (10) represents as briefly summarized in Table 6 below. Electrical double layer capacitance values could be calculated using any of Eqs. (11), (12), and (13) while percent inhibition efficiency, $\eta_{E}\left(\%\right)$, in the presence and absence of OGCIs is determined by Eq. (14) [78].

(11) $$C_{dl}={\left(AR_{ct}^{1-n}\right)}^{\frac{1}{n}}$$

(12) $$C_{dl}=A{\left(\omega_{\max}\right)}^{n-1}$$

(13) $$C_{dl}=\frac{1}{2\pi \omega_{\max}R_{ct}}$$

(14) $$\eta_{E}\left(\%\right)=\frac{R_{ct\left(i\right)}-R_{ct\left(o\right)}}{R_{ct\left(i\right)}}\times 100\%$$

where $\omega_{\max}$ represents maximum frequency of impedance imaginary quantity (rad s^−1^), *R*_*ct(i)*_ and *R*_*ct(o)*_ represent charge transfer resistance in the presence and absence of OGCI various concentrations respectively.

**Table 6:** Significance of *n* values on CPE nature.

n value	CPE nature (A)	Significane	Reference
0	Resistance	Metal-solution interface operates as a resistor.	[88]
1	Capacitance	Plane and homogeneous electrode surface with metal–solution interface behaving as a capacitor having regular surface.	[89]
−1	Inductance	Non-plane and heterogeneous electrode surface with metal–solution interface behaving as an inductor having irregular surface.	[90]
1/2	Warburg Impedance	A metal-solution interface acting as both capacitor and inductor.	[91]

In general, Table 7 summarizes significance/implication of changes in trends and variations in the values of parameters associated with techniques of measuring OGCIs efficiencies as observed in previous studies.

**Table 7:** Summary of significance of parameters variations obtained from methods of measuring OGCIs efficiencies as observed in previous studies.

Observation	Significance/Implication	Reference
**Weight Loss Measurement (WLM)**		
Increase in inhibition efficiency as OGCI concentration increases.	Adsorption of enough molecules of OGCI on the surface of metal at higher concentration causing higher surface coverage.	[57]
Inhibition efficiency of OGCI “A” in combination with small concentration of OGCI “B” is higher than the summation of inhibition efficiencies of OGCI “A” alone	Synergism parameter is greater than 1 which suggests better corrosion protection metallic specimens by OGCIs “A + B” than using OGCI “A” alone.	[77]
Inhibition performance of OGCI molecules decreased with increasing solution temperatures.	1. This resulted from increased in mobility of OGCI molecules which decreased existing interaction between metallic surface and OGCI molecules. 2. Rapid etching, molecular rearrangement and/or fragmentation and desorption of adsorbed OGCI molecules at higher temperature might decrease inhibition efficiency.	[88]
**Potentiodynamic Polarization (PDP)**		
Change of *E*_*corr*_ values to more negative values in different OGCI concentrations coupled with decrease in cathodic and anodic current density with increasing OGCI concentrations.	Adsorption of molecules of OGCI on sample surface, forming a protective metal surface. Cathodic polarization occurred. Anodic polarization occurs when anode potential shifts to positive direction. *E*_*corr*_ being >85 mV implies anodic or cathodic OGCI while *E*_*corr*_ displacement of <85 mV means mixed type OGCI.	[36, 92]
Increase in OGCI concentrations causing reduction of corrosion current density (*i*_*corr*_) with increase in inhibition efficiency (*IE*).	OGCI is effective in protecting metal in acidic medium solution.	[41]
Cathodic Tafel slope (β_c_) and anodic Tafel slope (β_a_) changed due to the addition of OGCI.	OGCI influences anodic and cathodic reactions.	[93]
Anodic and cathodic branches of Tafel plot shifted to lower values for all examined concentrations of OGCI added.	Organic constituents of OGCI inhibited both hydrogen evolution (cathodic reaction) and metal dissolution (anodic reaction) suggesting OGCI acted as mixed type.	[94]
**Electrochemical Impedance Spectroscopy (EIS)**		
Significant increase in *R*_*p*_ value as a result of adding inhibitor.	Charge transfer reaction retarded by inhibitors and corrosion occurring on metal surface with formation of protective film.	[95]
Reduction of *C*_*dl*_ values due to inhibitors molecules presence.	1. This resulted from local dielectric constant decrease and/or electrical double layer thickness increase. 2. Also, surface metal inhibition resulted from adsorption mechanism by water molecules replacement. 3. Increase in surface coverage by OGCIs causing Inhibition efficiency increase.	[96]
Imperfect semicircle obtained in Nyquist plots when concentration of OGCI increases in the solution.	This is attributed to metal surface imperfections and roughness called dispersing effect.	[97]
Phase angle values in Bode plot for inhibited metallic specimens higher than uninhibited specimen.	Surface becomes appreciably smooth due to protective film formation by OGCIs over metallic surface.	[81]
Increase in Nyquist plots diameter with increasing OGCIs concentration.	This indicates inhibitive film strengthening with decrease corrosion rate.	[98]
Nyquist plot contains a depressed semicircle for used solid electrode	This is linked to metal electrodes inhomogeneities and surface roughness.	[20]
Significant increase in *R*_*ct*_ in OGCIs molecules presence.	Adsorption of OGCIs molecules on metallic surface. High corrosion protection efficiency.	[99]
Decrease in Nyquist plots diameter with increasing solution temperature.	Corrosion inhibition rate is decreasing with increase in solution temperature.	[86]
Decrease in values of *R*_*ct*_ and *IE* as a result of increase in temperature.	Adsorbed OGCI molecules on metal surface subjected to desorption with continuity in increase of solution temperature.	[100]
Values of slope and phase angle deviating from ideal capacitive behavior of the electric double layer (slope = 1 and phase angle = -90^o^) in the Bode impedance and phase angle plots for inhibited and uninhibited metallic specimens.	This resulted from metallic surface inhomogeneity.	[101]

## 2 Main text

With reference to Fig. 2 depicting various sources of OGCIs, Tables 8, 9, and 10 summarize literatures consulted for drugs, ionic liquids and synthetic inhibitors as different OGCIs sources respectively used for testing various kinds of metallic materials in different corrosive media. Literatures consulted for plant extracts used as corrosion inhibitors are summarized in the supplementary table as part of supplementary material of this manuscript.

**Table 8:** Pharmaceutical drugs as OGCIs.

Class	Source/Origin	Medical Usage	OGCIs	Material and Tested Medium	Type	Reference(s)
Quinolones	Heterobicyclic aromatic compound quinoline (Obtained oily substance after quinine alkaline distillation).	UTI	Enofloxacin	Mild steel in NaCl solution	Mixed inhibitor	[102]
			Ofloxacin	Mild steel in HCl solution	Mixed inhibitor	[103]
			Ciprofloxacin	1. Stainless steel type 304 in NaCl solution. 2. Mild steel in HCl solution.	Mixed inhibitor	[104]
			Sparfloxacin	1. Mild steel in HCl solution. 2. Mild steel in H_2_SO_4_ solution	Adsorption	[105]
Macrolides	Streptomyces bacteria	STI and RTI	Erythromycin	Zinc in 0.01–0.04M H_2_SO_4_ solution	Adsorption	[106]
			Azithromycin	Zinc in H_2_SO_4_ solution	Adsorption	[107]
β-lactam antibiotics	Penicillins	STI, RTI and UTI	Penicillin V	1. Mild steel in H_2_SO_4_ solution	Adsorption	[108]
			Ampicillin	1. Aluminium in HCl solution. 2. Mild steel in H_2_SO_4_ solution	Adsorption	[109]
			Dicloxacillin	Aluminium 6063 in H_3_PO_4_ solution.	Mixed	[110]
			Amoxycillin	Aluminium and AA2024-T3 alloy in HCl solution	Adsorption	[111]
Tetracyclines	Metabolism or chemical modification of Streptomyces species	URTI and STD	Doxycycline	Cobalt-chromium alloy (Vitallium) and mild steel in KCl and HCl solutions.	Mixed	[112]
			Oxytetracycline	Cobalt-chromium alloy (Vitallium), stainless steel and Titanium in KCl and NaCl solutions	Mixed	[113]
Sulphonamides	SO_2_-NH_2_ moiety	CNSI, RTI, UTI, GITI	Sulfamethazine	Mild steel in HCl solution	Mixed	[114]
			Sulfacetamide	Carbon steel in HCl solution	Mixed	[115]
Aminoglycosides	-	UTI	Streptomycin	Mild steel in HCl solution	Mixed	[116]
Amphenicols	Phenylpropanoid	TF, SOI	Chloramphenicol	Mild steel in H_2_SO_4_ solution	Adsorption	[117]

Note: UTI = Urinary Tract Infections, STI = Soft Tissue Infections, RTI = Respiratory Tract Infections, URTI = Upper Respiratory Tract Infections, STD = Sexually Transmitted Diseases, CNSI = Central Nervous System Infections, GITI = Gastrointestinal Tract Infections, TF = Typhoid Fever, SOI = Superficial Ocular Infections.

**Table 9:** Ionic liquids as OGCIs.

Ionic Liquids Used as OGCIs	Material and Corrosive Medium	Characterization Technique	Observation	References
ImDC_18_Br and PyC_18_Br	Mild steel in 1M H_2_SO_4_	SEM-EDX, XRD, Mossbauer analyses	1. Acted as good OGCI. 2. Chemisorption adsorption mechanism. 3. Langmuir adsorption isotherm was obeyed. 4. Mixed type OGCI.	[118]
BMIC, HMIC, OMIC	Aluminum in 1M HCl	EIS, WL	1. Order of corrosion inhibition efficiency was OMIC˃HMIC˃BMIC. 2. Mixed type OGCIs. 3. Langmuir adsorption isotherm obeyed.	[119]
[BMIM]HSO_4_, [HMIM]HSO_4_ and [OMIM]HSO_4_	Copper in 0.5M H_2_SO_4_	EIS, PDP	1. Inhibition efficiency order of [OMIM]HSO_4_ > [HMIM]HSO_4_ > [BMIM]HSO_4_ was obtained. 2. Langmuir adsorption isotherm was obeyed. 3. Mixed type OGCIs.	[120]
EMID	Mild steel in 0.1M H_2_SO_4_	EIS, TFM	1. Decreased values of C_dl_. 2. Increased surface coverage. 3. Langmuir adsorption isotherm was obeyed.	[121]
[BMIM][BF_4_^−^] and [DMIM][BF_4_^−^]	Zinc in 1M HCl	GA, DFT	1. Good OGCIs. 2. Inhibition efficiencies increased as concentrations increased. 3. Physisorption adsorption mechanism. 4. Order of inhibition efficiency was [DMIM][BF_4_^−^]˃[BMIM][BF_4_^−^]. 5. Adsorption process followed Langmuir isotherm.	[122]
OPEIB	6061 Al-15 Alloy in 0.1M H_2_SO_4_ solution	EIS, PDP, SEM, EDX	1. Acted well as good OGCI. 2. Inhibition efficiency increases with concentration. 3. Temkin adsorption isotherm obeyed.	[123]
TDPB	Aluminum in 1M HCl	WL,EIS	1. OGCI acted as cathodic type for acidic aluminum corrosion. 2. Corrosion inhibition by adsorption on metallic surface. 3. Langmuir adsorption isotherm was obeyed.	[124]
BMIC and [BMIM]HSO_4_	Mild steel in 1M HCl	EIS, WL	1. Inhibition efficiency of [BMIM]HSO_4_ higher than BMIC. 2. Mixed type OGCI. 3. Adsorption obeyed Langmuir.	[125]
[OMIM]Br and AOIM]Br	Mild steel in 0.5M H_2_SO_4_	WL, EIS, SEM	1. Acted as good OGCIs. 2. Adsorption obeyed El-Awady thermodynamic–kinetic model. 3. Ionic liquids acted slightly as cathodic type inhibitors.	[126]
IL1 and IL2	CuSn_8_P and steel 100Cr_6_ in water	ICP-OES, SEM, EDX, XPS	-	[127]
PImC12, PImC8 and PImC4	Aluminum alloy AA6061 in 0.1–1.0M H_2_SO_4_ solution	WL, PDP, ICP-OES	1. Order of inhibition efficiency was PImC12˃PImC8˃PImC4 2. Langmuir adsorption isotherm obeyed. 3. Mixed type OGCIs.	[128]

Note: ImDC_18_Br = 1,3 dioctadecylimidazoliumbromide, PyC_18_Br = N-octadecylpyridiniumbromide, EMID = 1-ethyl-3-methylimidazolium dicyanamide, BMIC = 1-butyl-3-methylimidazolium chlorides, [BMIM]HSO_4_ = 1-butyl-3-methylimidazolium hydrogen sulfate, [OMIM]Br = 1-octyl-3-methylimidazolium bromide, [AOIM]Br = 1-allyl-3-octylimidazolium bromide, HMIC = 1-hexyl-3-methylimidazoliumchlorides, OMIC = 1-octyl-3-methylimidazoliumchlorides, OPEIB = 1,3-bis(2-oxo-2-phenylethyl)-1H-imidazol-3-ium bromide, PImC12 = poly(1-vinyl-3-dodecylimidazolium, PImC8 = poly(1-vinyl-3-octylimidazolium), PImC4 = poly(1-vinyl-3-butylimidazolium), TDPB = Tetradecylpyridiniumbromide, [BMIM]HSO_4_ = 1-butyl-3-methylimidazolium hydrogen sulfate, [HMIM]HSO_4 =_ 1-hexyl-3-methylimidazolium hydrogen sulfate, [OMIM]HSO_4_ = 1-octyl-3-methylimidazolium hydrogen sulfate, IL1 = (2-hydroxyethyl)-trimethyl-ammonium, IL2 = Butyltrimethyl-ammonium, [BMIM][BF_4_^−^] = 1-butyl-3-methylimidazolium tetrafluoroborate, [DMIM][BF_4_^−^] = 1-decyl-3-methylimidazolium tetrafluoroborate.

**Table 10:** Synthetic OGCIs.

OGCIs Source	Synthesis Methodology	MaterialTested/Solution Used	Characterization	Findings	References
*o,m,p-*decanoyl thiourea derivatives	Mixed substitution and addition reaction using decanoyl chloride, ammonium thiocyanate and 2-aminopyridine in acetone solution for 10 mins.	Mild steel in 0.1 M H_2_SO_4_	FTIR, ^1^H and^13^C NMR	1. Compound D3 of the derivatives possessed highest efficiency. 2. Compounds Corrosion inhibition efficiencies affected by N atom at *ortho, meta* and *para* position effects in pyridine chemical structure.	[129]
Isoxazolidine derivatives of aldehyde	Nitrone cycloaddition reaction	Mild Steel in 1M HCl, 0.5M H_2_SO_4_, CO_2_-saturated 0.5 M NaCl	GM, LPR, TEM, EIS, ST	1. Inhibitor molecules primarily acted as anodic inhibitors. 2. Adsorption of inhibitors on metal surface was due to physisorption and chemisorption. 3. Surface tension revealed formation of film at the surface of metal by inhibitor molecules. 4. Inhibitor molecules fitted well using Temkin isotherm in both acids. 5. Langmuir isotherm performed excellently in CO_2_-saturated saline media.	[130]
Hydroxyethyl-imidazoline derivatives based on coffee oil	-	Carbon steel in CO_2_-saturated emulsion at 50 °C.	EIS	1. Inhibitor decreased corrosion rate by over 99.9%. 2. Unprotected sites was linked to electrostatic repulsion forces between the negative charges and insufficient added concentration to form protective film.	[131]
Ammonium surfactants based polyethylene glycol	Reflux of Dibromoethanoate polyethylene glycol with N, Ndiethyl aniline in ethyl alcohol for 12 h.	steel in 1.0M HCl	WL, EIS, PDP	1. Inhibition efficiency of 94% was achieved at highest temperature of 55 °C. 2. Physicochemical adsorption mechanism. 3. Mixed type OGCI. 4. Langmuir isotherm model was obeyed.	[132]
Sodium lignosulfonate	-	Zinc sheets in 0.01M HCl	WL, EIS, PDP	1. Acted as good synthetic OGCI.	[133]
2-(coumarin-4-yloxy)acetohydrazide	Reflux of methyl bromoacetate with 4-hydroxycoumarin in anhydrous acetone in the presence of anhydrous potassium carbonate.	Mild steel in 1.0M HCl	WL, FTIR, DFT, NMR	1. 94.7% corrosion inhibition efficiency was obtained. 2. Langmuir adsorption isotherm obeyed.	[134]
2-Amino 5-Oleyl-1,3,4-Thiadiazol	Cyclization of oleic acid	Mild steel in 1M HCl	FTIR, NMR, PDP, EIS	1. Acted as good corrosion inhibitor for the medium. 2. Presence of molecule active site on Nitrogen atom in the heterocyclic ring.	[135]
3-nitrobenzoic acid	-	Mild steel in 0.1M H_2_SO_4_	WL, PDP, EIS, SEM, FTIR	1. Exothermic and spontaneous adsorption. 2. Cathodic-type inhibitor. 3. Langmuir adsorption model. 4. WL, PDP and EIS revealed inhibition efficiencies of 87.15, 90.51 and 99.40% respectively at inhibitor's concentration of 0.01 M.	[136]
Aldehyde isoxazolidine derivatives	Nitrone cycloaddition reaction.	Mild steel in 1M HCl, 0.5M H_2_SO_4_, and CO_2_-saturated 0.5M NaCl	GM, PDP, SEM, EIS	1. *p-*9-[hexahydropyrrolo (1,2-b)isoxazol-2-yl]nonyloxybenzaldehyde performed best as compared to others. 2. Anodic inhibitors type. 3. Temkin adsorption isotherm obeyed in both acidic media and Langmuir adsorption isotherm in CO_2_-saturated saline media.	[137]

### 2.1 Industrial applications of OGCIs

Industrial applications of corrosion inhibitors from greeners can be found in petroleum production, steel pipelines making industry, refrigerating industry, automobile, paint industry, acid producing companies and so on. Table 11 sumarizes industrial applications of OGCI with active functional groups responsible for each application.

**Table 11:** Industrial applications of OGCIs.

Industrial Application	Active Functional Groups/Complexes/Ingredients	Inhibitor Source from Greener	% *IE*	How it works/How to solve the problem	Side Effects	References
Petroleum Production	Pyrocatechol		−14	Petroleum industries are characterized with wet corrosion of materials as a result of aqueous phase existence which may contain H_2_S, CO_2_ and Cl^−^. The injection of these film-forming long-chain nitrogenous inhibitors anchors to metal surface via existing polar group. The non-polar tail extends out vertically such that physisorption of hydrocarbons on them increases thickness of the film coupled with hydrophobic barrier effectiveness to prevent corrosion.	Emulsification occurs which leads to foaming as a result of inhibitors being interfacial in nature.	[138]
	4-Methylpyrocatechol		84			
	4-*n*-butylpyrocatechol		93			
	4-n-hexylpyrocatechol		96			
Steel Pipelines Internal Corrosion	Galactose and mannose	Guar gum	86	Flow-induced corrosion and erosion-corrosion are influenced by high flow rates of multiphase fluids in steel pipelines. At low flow rates, corrosion pitting occurs due to sediments formation at the bottom. The inhibitors being mixed type prevent corrosion by physical adsorption, chemisorption and film formation. Also, pigging of steel pipelines is employed to avoid internal corrosion.	Due to mixed reaction, unwanted products and intermediates may be formed in the course causing formation of unwanted sediments	[62, 63]
	Iridoid glucoside and Dibenzo-α-pyrone	Leaf extract of *Anthocleista Djalonesis*	97			
	Flavonoids and terpenoids	*Ginkgo biloba leave extracts*	98			
Automobiles	Phosphates and silicates	Rice husk extract	92	The inhibitors dissolve in antifreeze to prevent internal corrosion caused by coolants, aeration, temperature, flow and so on. External corrosion is controlled by mixing additives such as grease, wax resin, metalloorganic and asphaltic compounds that enhance film formation on metal surface.	Foaming due to emulsification occurs	[59]
	Fatty acids, phosphonates and sulfonates	*Diospyros* Kaki L.f husk extracts	95			
		Oil palm fond				
Paint Industry	Calcium plumbate, lead azelate and lead suboxide	-		Displacement of water by polar compounds in inhibitors occurs after which they arrange themselves with hydrophobic ends facing the environment. The augmentation of coatings bonding on the surface of metals occurs aftermath.	Intermediate pigments may be formed.	-
Water Transmission Industry	Phosphates, amines volatiles (cyclohexylamine, morphine)	Tobacco extract	78.3	Inhibitors anchors to the metal using their polar group which increases film thickness and hydrophobic barrier effectiveness for corrosion inhibition.	Interaction between organic inhibitor and water makes the water unsuitable for domestic usage in most cases.	[139]
Refrigerating Industry	Benzotriazole	*Anthocleista djalonesis*	88	Galvanic corrosion evolves due to increase in dissolved mineral salt content as evaporation proceeds with the presence of several dissimilar metals and non-metals. Inhibitors control corrosion by films formation that inhibits anodic metal dissolution reaction and cathodic poisoning.	-	[62, 140]
	p-hydroxybenzoic acid, and vanillic acid	Oil palm frond	67.8			
Building Construction	Phosphate ion	-	-	When mixed with cement, durability of reinforced concrete structures is improved.	-	[141]
Boiler	Ammonia, alkanol, Cyclohexylamine and Morpholine	-	-	Corrosion attack of pipes prevented by solubilization of limescale.	-	[24]

### 2.2 Kinetics of corrosion modelling

#### 2.2.1 Anodic modelling

In order to model kinetics of corrosion at the anode, the following assumptions are made: (1) anodic corrosion current density was used for Fe^2+^ ion boundary condition at anode (2) Anodic corrosion current density accounts for Fe^2+^ ion generation via electrochemical reactions at the surface of metal as the source term (3) Zero concentration of Fe^2+^ ion is applied at cathode due to scale formation (4) Fe^2+^ ion concentration in the shielded solution is the same as bulk solution in chemical equilibrium and (5) H^+^ ($C_{H^{+}}$) and CO_2_ ($C_{CO_{2}}$) surface concentrations enhance the rate of corrosion via exchange current density. Thus, the anodic electrochemical reaction is given as Eq. (15) [5]:

(15) Fe → Fe^2+^ + 2e^−^

The anodic corrosion current density is calculated using Eq. (16) (Tafel's law):

(16) $$i_{Fe^{2+}}=i_{0,Fe^{2+}}.10^{\frac{\phi_{a}-\phi_{rev,\;Fe^{2+}}}{b_{Fe^{2+}}}}$$

where $i_{Fe^{2+}}$ = current density of iron oxidation (A/m^2^), $i_{0,Fe^{2+}}$ = exchange current density of iron oxidation (A/m^2^), $\phi_{a}$ = anodic potential (V), $\phi_{rev}$ = reversible potential of iron oxidation (V) and b = Tafel slope of oxidation (V).

The iron oxidation exchange current density ($i_{0,Fe^{2+}}$) in Eq. (16) is determined from Eq. (17) thus [142]:

(17) $$i_{0,Fe^{2+}}=i_{0,ref}{\left(\frac{C_{H^{+}}}{C_{H^{+},ref}}\right)}^{a_{1}}.{\left(\frac{C_{CO_{2}}}{C_{CO_{2},ref}}\right)}^{a_{2}}.e^{\frac{-\Delta H}{R}\left(\frac{1}{T}-\frac{1}{T_{ref}}\right)}$$

where $i_{0,ref}$ = reference exchange current density (A/m^2^), $C_{H^{+}}$ = Surface concentration of hydrogen ion (mol/L), $C_{H^{+},ref}$ = Reference hydrogen ion concentration (mol/L), $C_{CO_{2}}$ = Surface concentration of carbondioxide (mol/L) and $C_{CO_{2},ref}$ = reference carbondioxide concentration (mol/L), *ΔH* = change in enthalpy (kJ/mol), *R* = gas constant (J/mol·K), *T* = solution temperature (/K) and *T*_*ref*_ = reference temperature (/K).

The mass flux of Fe^2+^ at anode ($J_{Fe^{2+}}$) is determined by using Eq. (18) [143]:

(18) $$J_{Fe^{2+}}=\frac{i_{Fe^{2+}}}{n_{Fe^{2+}}F}$$

where $J_{Fe^{2+}}$ = mass flux of Fe^2+^ at anode (mol/m^2^·s), $i_{Fe^{2+}}$ = current density of iron oxidation (A/m^2^), F = Faraday's constant (C/mole) and $n_{Fe^{2+}}$ = number of moles of Fe^2+^ (moles).

#### 2.2.2 Cathodic modelling

The derivation of equations governing kinetics of corrosion at the cathode is based on the assumption that oxygen and water reduction in the system is negligible such that the two cathodic reactions are stated as Eqs. (19) and (20) [144]:

(19) 2H^+^ + 2e^−^ → H_2_

(20) 2H_2_CO_3_ + 2e^−^ → H_2_ + 2HCO_3_^−^

A general form used in the calculation of H^+^ reduction partial cathodic corrosion current densities and H_2_CO_3_ reduction is stated as Eq. (21) [142]:

(21) $$i_{c}=i_{0}.10^{-\frac{\phi_{c}-\phi_{rev}}{b}}.\eta_{Scale}$$

where $i_{c}$ = current density of any cathodic reaction (A/m^2^), $i_{0}$= cathodic reaction exchange current density (A/m^2^), $\phi_{c}$ = cathodic potential (V), $\phi_{rev}$ = cathodic reaction reversible potential (V), b = cathodic reaction Tafel slope (V) and $\eta_{Scale}$ = Scale factor at cathode.

The exchange current densities of H^+^ and H_2_CO_3_ reduction at cathode are determined using Eq. (17). The electric field in the solution is governed by Poisson's equation is stated as:

(22) $$\nabla^{2}\phi =-\frac{F}{\varepsilon }\sum_{i=1}^{n}z_{i}c_{i}$$

where ε = dielectric constant and ϕ = potential (V).

For electro-neutrality condition in the solution, Eq. (22) reduces to:

(23) $$\sum_{i=1}^{n}z_{i}c_{i}=0$$

Thus, Eq. (23) reduces to

(24) $$\nabla^{2}\phi =0$$

#### 2.2.3 Electrochemical modelling

Assuming corrosion rate is governed only by electrochemical reaction, total anodic reaction current density is used in determining corrosion rate of CO_2_ stated as [145]:

(25) $$CR=\frac{i_{a}M_{w,Fe}}{\rho_{Fe}nF}$$

where CR = corrosion rate (mm/a), *i*_*a*_ = anodic current density (A/m^2^), *M*_*w,Fe*_ = atomic mass of iron (kg/mol), $\rho_{Fe}$ = density of iron (kg/m^3^), *n* = number of moles of electrons involved in iron oxidation (2 mol_e_/mol) and *F* = Faraday's constant.

The current density for iron dissolution is obtained by using Eq. (26) stated as [146]:

(26) $$i_{a,Fe}=i_{o,Fe}\times 10^{\left[\frac{\alpha_{Fe}F\left(E_{corr}-E_{rev,Fe}\right)}{RT}\right]}$$

The Tafel slope of Iron oxidation $b_{Fe}$ as defined as [147]:

(27) $$b_{Fe}=\frac{RT}{\alpha_{Fe}F}$$

where R= ideal gas constant (J/mol·K), T = Temperature (K), F= Faraday's constant and $\alpha_{Fe}$ = iron dissolution constant.

Thus, Eq. (26) reduces to

(28) $$i_{a,Fe}=i_{o,Fe}\times 10^{\left[\frac{\left(E_{corr}-E_{rev,Fe}\right)}{b_{Fe}}\right]}$$

where $i_{a,Fe}$ = current density for Iron dissolution (A/m^2^), $i_{o,Fe}$= exchange current density of iron oxidation (A/m^2^), *E*_*corr*_ = corrosion potential (V), *E*_*rev,Fe*_ = reversible potential of iron oxidation (V), $b_{Fe}$ = Tafel slope of iron oxidation (V).

The current density of any cathodic reaction is calculated thus [148]:

(29) $$\frac{1}{i_{c}}=\frac{1}{i_{ct}}+\frac{1}{i_{\lim}}$$

where $i_{c}$ = cathodic reaction current density (A/m^2^), $i_{ct}$ = charge transfer current density component (A/m^2^) and $i_{\lim}$ = limiting current density component (A/m^2^).

The charge transfer current density of cathodic reactions ($i_{ct}$) is determined by using [149]:

(30) $$i_{ct}=i_{o}.10^{-\frac{\eta }{b_{c}}}$$

where $i_{o}$ = exchange current density of cathodic reactions (A/m^2^), $\eta =E-E_{rev}$ is the overpotential (V), E = potential (V), $E_{rev}$= reversible potential (V) and $b_{c}$ = cathodic Tafel Slope (V/decade).

The limiting current is determined from the mass transfer limitation for the case of H^+^ reduction. Thus,

(31) $${i_{\lim\left(H^{+}\right)}^{d}=k_{m}F.\left[H^{+}\right]}_{b}$$

where $i_{\lim\left(H^{+}\right)}^{d}$ = diffusion limiting current density (A/m^2^), $k_{m}$ = mass transfer coefficient of corrosive species (m/s), ${\left[H^{+}\right]}_{b}$ = bulk hydrogen ion concentration (mol/m^3^) and F = Faraday constant (96,490 C/equiv.)

Suppose there is restriction of carbonic acid reduction due to CO_2_ hydration reaction rate being very slow, limiting current density ($i_{\lim\left(H_{2}CO_{3}\right)}^{r}$) is calculated as [150]:

(32) $${{i_{\lim\left(H_{2}CO_{3}\right)}^{r}=F.\left[CO_{2}\right]}_{b}.\left(D_{H_{2}CO_{3}}K_{hyd}k_{hyd}^{f}\right)}^{0.5}$$

where ${\left[CO_{2}\right]}_{b}$ = bulk concentration of dissolved carbon dioxide (mol/m^3^), $D_{H_{2}CO_{3}}$ = diffusion coefficient of H_2_CO_3_ in water (m^2^/s), $K_{hyd}$ = equilibrium constant for CO_2_ hydration reaction and $k_{hyd}^{f}$ = forward reaction rate constant for the CO_2_ hydration reaction (s^−1^).

A theoretical flow multiplier *f* for Eq. (32) which takes into account the flow effect on the chemical reaction limiting current is calculated by using [145]:

(33) f=1+e−2δmδr1−e−2δmδr

where $\delta_{m}$ = mass transfer thickness (m) and $\delta_{r}$ = reaction layer thickness (m) whose values are determined by using Eqs. (34) and (35) respectively.

(34) $$\delta_{m}=\frac{D_{H_{2}CO_{3}}}{{k_{m,}}_{H_{2}CO_{3}}}$$

and

(35) $$\delta_{r}=\sqrt{\frac{D_{H_{2}CO_{3}}K_{hyd}}{k_{hyd}^{f}}}$$

### 2.3 Rate modelling of corrosion types inhibition using OGCIs

#### 2.3.1 Pitting corrosion

The risk of pitting corrosion could be increased under stagnant conditions in which corrosive microenvironments are established on the surface. The accumulation of stagnant electrolyte at the bottom of pipes, tubes and tanks could be prevented by both drying and ventilation. The buildup of local highly corrosive conditions could also be prevented through agitation [151]. Pitting corrosion rate, defined as Fe^2+^ ion mass flux leaving the metal surface, can be determined using Eq. (36) based on the following assumptions: (1) pitting corrosion results into the removal of Fe^2+^ ion from the metal surface by diffusion and electro-migration, and (2) Fe^2+^ ion distribution in the solution is governed by Fick's second law. Thus, Fe^2+^ flux can be solved using Nernst-Planck equation [152]:

(36) $$J_{Fe^{2+}}=-D_{Fe^{2+}}\nabla C_{Fe^{2+}}-\frac{Fz_{Fe^{2+}}}{ℜT}D_{Fe^{2+}}C_{Fe^{2+}}\nabla \phi$$

where J = mass flux (mol/m^2^·s), D = diffusivity (m^2^/s), C = concentration (mol/L), F = Faraday's constant (C/mole), z = valence (mole/mol), ℜ = ideal gas constant (J/mol·K), T = absolute temperature (K) and ϕ = electric potential (V).

The pitting corrosion rate is determined after computing the distributions of Fe^2+^ ion and electrical field in the solution such that change in concentration with time $\left(\frac{\partial C}{\partial t}\right)$ is determined thus [153]:

(37) $$\frac{\partial C}{\partial t}=-\nabla \left(J\right)+R$$

where R = source term of chemical reactions in the solution.

#### 2.3.2 Stress corrosion cracking (SCC)

Stress corrosion cracking in metallic materials had been discussed previously [5]. The outstanding models for predicting stress corrosion cracking rate are active path dissolution and film rupture model; and hydrogen assisted cracking model. Active path dissolution occurs as a result of accelerated corrosion within a narrow path having higher corrosion susceptibility in comparison with overall material or structure. On the other hand, hydrogen assisted cracking involves entrapment of hydrogen atoms onto metal crystal structure and the subsequent local cracking resulting from local pressure build up [154].

*Active path dissolution and film rupture model*: For this, crack growth rate or crack velocity ($\dot{a}$ ) is given as [155]:

(38) $$\dot{a}=\frac{da}{dt}=A{\left({\dot{\varepsilon }}_{ct}\right)}^{n}$$

where A and *n* = constants related to material and environmental composition at the crack tip and ${\dot{\varepsilon }}_{ct}$ = crack tip strain.

The pure oxidation dissolution Faradic equation is stated as [156]:

(39) $$\dot{a}=\frac{da}{dt}=\frac{M}{z\rho F}i_{t}^{a}$$

where M = atomic weight, z = oxidation number, ρ = density (kg/m^3^), F= Faraday constant and $i_{t}^{a}$ = anodic current density at time t.

The anodic current density at time t is given as [157]:

(40) $$i_{t}^{a}=i_{o}^{a}{\left(\frac{t_{o}}{t}\right)}^{n}$$

where $i_{o}^{a}$ = base metal dissolution rate parameter, $t_{o}$ = repassivation time scaling parameter (secs or min or hr).

Substituting Eq. (40) into (39) gives

(41) $$\dot{a}=\frac{da}{dt}=\frac{M}{z\rho F}i_{o}^{a}{\left(\frac{t_{o}}{t}\right)}^{n}$$

If $a^{*}=\frac{M}{z\rho F}i_{o}^{a}$, Eq. (41) reduces to

(42) $$\dot{a}=\frac{da}{dt}=a^{*}{\left(\frac{t_{o}}{t}\right)}^{n}$$

Integrating Eq. (42) over time from $t_{o}\to t_{f}$ and averaging the stress corrosion cracking (SCC) growth rate over $t_{f}-t_{o}$, the average SCC growth rate is expressed as:

(43) $$\bar{\dot{a}}=\frac{1}{t_{f}-t_{o}}\int \dot{a}\;dt=\frac{a^{*}}{1-n}{\left(\frac{t_{o}}{t_{f}}\right)}^{n}$$

where $t_{f}$ = time per oxide fracture event (secs or min or hr) defined as:

(44) $$t_{f}=\frac{\varepsilon_{f}}{{\overset{\cdot }{\varepsilon }}_{ct}}$$

where $\varepsilon_{f}$ = oxide film rupture strain rate and ${\overset{\cdot }{\varepsilon }}_{ct}$ = crack tip strain rate.

Assuming ${\overset{\cdot }{\varepsilon }}_{ct}$ and $t_{f}$ are constant and independent of time, Eq. (44) can be substituted in Eq. (43) to give the average SCC growth rate as:

(45) $$\bar{\overset{\cdot }{a}}=\frac{a^{*}}{1-n}{\left(\frac{t_{o}}{\varepsilon_{f}}\right)}^{n}{\left({\overset{\cdot }{\varepsilon }}_{ct}\right)}^{n}$$

*Hydrogen embrittlement*: The related stress corrosion cracking model is based on the assumption that crack advance occurred due to hydrogen assisted creep fracture (HACF) of hydrogen embrittled grain boundaries. Thus, stress corrosion crack growth rate is expressed as [154]:

(46) a·=rct=rc(εfε·cfz)=rcε·cfzεf

where $r_{c}$ = radius of fracture zone in front of crack tip (mm), ${\overset{\cdot }{\varepsilon }}_{cfz}$ = strain rate in creep fracture zone and $\varepsilon_{f}$ = critical fracture strain.

The critical fracture strain ($\varepsilon_{f}$) can be stated as [158]:

(47) $$\varepsilon_{f}=\varepsilon_{f_{o}}{\left(\frac{C_{o}}{C_{gb}}\right)}^{\frac{1}{2}}$$

where $C_{gb}$ = grain boundary hydrogen concentration, $\varepsilon_{f_{o}}$ = fracture strain at a reference grain boundary hydrogen concentration, $C_{o}$ = reference grain boundary hydrogen concentration.

Substituting Eq. (47) into Eq. (46) gives

(48) $$\overset{\cdot }{a}=\frac{r_{c}{\overset{\cdot }{\varepsilon }}_{cfz}}{\varepsilon_{f_{o}}}{\left(\frac{C_{gb}}{C_{o}}\right)}^{\frac{1}{2}}$$

#### 2.3.3 H_2_S (sour) corrosion

H_2_S corrosion of mild steel proceeds predominantly through a solid state reaction as a result of dense, very thin and protective non-stoichiometric iron sulphide film formation called mackinawite [5, 159]. Assuming H_2_S corrosion is controlled by mass transfer resulting from the presence of mackinawite layers and the liquid boundary layer, H_2_S flux through the mass transfer boundary layer is expressed as [160]:

(49) $$J_{H_{2}S}=k_{m\left(H_{2}S\right)}\left(c_{H_{2}S}-c_{o\left(H_{2}S\right)}\right)$$

The flux of H_2_S through the porous outer mackinawite layer is calculated as:

(50) $$J_{H_{2}S}=\frac{D_{H_{2}S}\varepsilon \psi }{\delta_{os}}\left(c_{o\left(H_{2}S\right)}-c_{i\left(H_{2}S\right)}\right)$$

The flux of H_2_S through the inner mackinawite film is determined by using:

(51) $$J_{H_{2}S}=A_{H_{2}S}In\left(\frac{c_{i\left(H_{2}S\right)}}{c_{s\left(H_{2}S\right)}}\right)$$

From Eq. (49),

(52) $$c_{o\left(H_{2}S\right)}=c_{H_{2}S}-\frac{J_{H_{2}S}}{k_{m\left(H_{2}S\right)}}$$

From Eq. (50),

(53) $$c_{o\left(H_{2}S\right)}=c_{i\left(H_{2}S\right)}+\frac{J_{H_{2}S}\delta_{os}}{D_{H_{2}S}\varepsilon \psi }$$

At steady state, the fluxes through different layers are equal to each other. Equating Eq. (52) and Eq. (53) and making $c_{i\left(H_{2}S\right)}$ the subject, we have

(54) $$c_{i\left(H_{2}S\right)}=c_{H_{2}S}-J_{H_{2}S}\left[\frac{1}{k_{m\left(H_{2}S\right)}}+\frac{\delta_{os}}{D_{H_{2}S}\varepsilon \psi }\right]$$

The corrosion rate caused by H_2_S in metallic materilas is obtained by substituting Eq. (54) into Eq. (51). Thus,

(55) $$CR_{H_{2}S}=A_{H_{2}S}In\frac{c_{b,H_{2}S}-J_{H_{2}S}\left[\frac{1}{k_{m\left(H_{2}S\right)}}+\frac{\delta_{os}}{D_{H_{2}S}\varepsilon \psi }\right]}{c_{s,H_{2}S}}$$

where $CR_{H_{2}S}$ = corrosion rate caused by H_2_S (mol/m^2^s), $A_{H_{2}S}$ = constant for solid state diffusion, $c_{b,H_{2}S}$ = concentration of H_2_S in bulk solution (mol/m^3^), $c_{s,H_{2}S}$ = concentration of H_2_S at steel surface (mol/m^3^), $J_{H_{2}S}$ = flux of H_2_S at different mackinawite layer (mol/m^2^·s), $k_{m\left(H_{2}S\right)}$ = mass transfer coefficient of H_2_S in liquid boundary layer (m/s), $\delta_{os}$ = outer scale thickness (m), $D_{H_{2}S}$ = diffusion coefficient of H_2_S in water (m^2^/s), ε = outer mackinawite scale porosity and ψ = outer mackinawite scale tortuosity.

### 2.4 OGCIs adsorption isotherms

Adsorption isotherms play key role in giving detailed information about existing interaction between molecules of OGCIs and metal surface [161] in order to prevent the dissolution reaction of such metal in the corrosive medium. Influencing factors on adsorption process using OGCIs include: (1) structure of OGCIs compounds (2) types of corrosive media under examination (3) nature of surface-charged metals (4) electronic characteristics of metal surface and (5) charge distribution in the molecules of OGCIs [162, 163]. The values obtained from the simulation of existing isotherm models relating surface coverage (*θ*) and OGCI concentration together describe the most suitable adsorption isotherm for the process. Prominent OGCIs adsorption isotherms have been stated to be Langmuir, Temkin, Frumkin, Freudlich, Virial Parson and Bockris-Swinkels isotherms [164] which are summarized in Table 12 with their verification plots and significance of values obtained. The most suitable adsorption isotherm that best describes adsorption nature of OGCI on examined metal surface would give a correlation coefficient (*R*^*2*^) value that is very close to unity or equal to 1.

**Table 12:** OGCIs adsorption isotherms models and significance of values obtained.

Isotherm	Model	Plot	Significance of values	Reference
Temkin	$\theta =\left(\frac{1}{f}\right)InK.C_{OGCI\;Conc}$	θ vs $\log C_{OGCI\;conc}$	If *f* = 0 (no interaction), *f* = +ve (attraction) and *f* = −ve (repulsion) between OGCI molecules and metal surface	[165]
Virial Parson	$\theta .e^{2f\theta }=K_{ads}.C_{OGCI\;Conc}$	θ vs $\log\left(\frac{\theta }{C_{OGCI\;conc}}\right)$	1. If *f* = 0 (no interaction), *f* = +ve (attraction) and *f* = −ve (repulsion) between OGCI molecules and metal surface. 2. If larger value is obtained for *K*_*ads*_ (5×10^−3^ − 20×10^−3^M^−1^), it is an indication of strong adsorption capacity of OGCI attributed to abundant *p*-electron in conjugated double or triple bonds between OGCI and vacant *d*-orbital of metal specimen.	[166]
Langmuir	$\frac{\theta }{\left(1-\theta \right)}=K_{ads}.C_{OGCI\;conc}$	$\frac{\theta }{\left(1-\theta \right)}$ vs $\log C_{OGCI\;conc}$	Smaller value for *K*_*ads*_ implies weak adsorption capacity of OGCI.	[167]
Freudlich	$\log\;\theta =\log K_{ads}+n\;\log C_{OGCI\;Conc}$	logθ vs $\log C_{OGCI\;conc}$	1. A value of n > 1 implies a favourable adsorption of OGCI molecules on metal surface. 2. Larger value of *K*_*ads*_ implies strong adsorption capacity of OGCI.	[168]
Bockris-Swinkels	$\frac{\theta }{{\left(1-\theta \right)}^{n}}.{\left[\theta +n\left(1-\theta \right)\right]}^{\frac{n-1}{n^{n}}}=C_{OGCI\;Conc}.e^{\frac{-K_{ads}}{55.4}}$	$\frac{\theta }{\left(1-\theta \right)}$ vs $\log C_{OGCI\;conc}$	1. A value of n < 1 implies a unfavourable adsorption of OGCI molecules on metal surface. 2. Smaller value of *K*_*ads*_ implies weak adsorption capacity of OGCI.	[169]
Frumkin	$\left[\frac{\theta }{\left(1-\theta \right)}\right]e^{f\theta }=K_{ads}.C_{OGCI\;Conc}$	θ vs $\log C_{OGCI\;conc}$	1. If *f* = 0 (no interaction), *f* = +ve (attraction) and *f* = −ve (repulsion) between OGCI molecules and metal surface	[170]

Note: *θ* = Surface coverage, *C*_*OGCI Conc*_ = Concentration of bulk OGCI (mM), *K*_*ads*_ = Adsorption equilibrium constant (mol^−1^dm^3^ or M^−1^), *f* = OGCI interaction parameter, *n* = number of H_2_O molecules replaced per OGCI molecule and *K* = Constant.

### 2.5 OGCIs adsorption thermodynamics

The consideration of thermodynamics studies in the OGCIs adsorption reveals the significance of Gibbs free energy of adsorption $\left(\Delta G_{ads}^{o}\right)$, enthalpy of OGCIs adsorption $\left(\Delta H_{ads}^{o}\right)$, entropy of OGCIs adsorption $\left(\Delta S_{ads}^{o}\right)$ and apparent activation energy (*E*_*a*_) of the process. The value of adsorption equilibrium constant (*K*_*ads*_) obtained from the best fitted isotherm tabulated in Table 8 is used to calculate $\Delta G_{ads}^{o}$ by using Eq. (56) [171]. The adsorption heat, $\Delta H_{ads}^{o}$ can be calculated using any of Van't Hoff equation stated as Eq. (57) [172]. The entropy of OGCI adsorption, $\Delta S_{ads}^{o}$ can be calculated using Eq. (58) [95]. Also, enthalpy $\left(\Delta H_{a}\right)$ and entropy $\left(\Delta S_{a}\right)$ of activation for the corrosion process can be calculated from the results obtained from temperature studies via Eq. (59) such that a plot of $\frac{\log\;CR}{T}$ against $\frac{1}{T}$ gives a slope of $\left(\frac{-\Delta H}{2.303R}\right)$ and intercept of $\left(\log\left(\frac{R}{nh}\right)+\frac{\Delta S}{2.303R}\right)$ which enhance the computation of $\Delta H_{a}$ and $\Delta S_{a}$ [120]. However, many corrosion studies have shown that corrosion rate increases as temperature increases which was being justified by the Arrhenius equation stated as Eq. (60) [90]. Table 13 summarizes the significance of values of thermodynamic parameters on adsorption of OGCIs on metals.

(56) $$\Delta G_{ads}^{o}=-RTIn\left(55.5K_{ads}\right)$$

(57) $$InK_{ads}=\left(\frac{-\Delta H_{ads}^{o}}{RT}\right)+\text{constant}$$

(58) $$\Delta G_{ads}^{o}=\Delta H_{ads}^{o}-T\Delta S_{ads}^{o}$$

(59) $$\frac{\log\;CR}{T}=\log\left(\frac{R}{Nh}\right)+\frac{\Delta S_{a}}{2.303R}-\frac{\Delta H_{a}}{2.303RT}$$

(60) $$\log\;CR=\log\;A-\frac{E_{a}}{2.303RT}$$

where *CR* = Corrosion rate (mm/yr), *N* = Avogadro's number (6.02 × 10^23^ mol^−1^), *h* = Plank's constant (6.63 × 10^−34^ m^2^ kg·s^−1^), *R* = Gas constant (8314 J mol^−1^ K^−1^), *T* = Absolute temperature (°K), *E*_*a*_ = Activation energy (kJ mol^−1^) and *A* = Pre-exponential factor.

**Table 13:** Significance of values of thermodynamics parameters on adsorption of OGCIs on metals.

Thermodynamics parameter	Significance of values on adsorption process	Reference
$\Delta G_{ads}^{o}$	If negative value is obtained, it implies the adsorption process is spontaneous with formation of stable protective OGCI layer.	[173]
	$\Delta G_{ads}^{o}$ ≤ −20 kJ/mol indicates electrostatic interaction existence between charged molecules of OGCI and charged metal simply called physisorption.	[174]
	$\Delta G_{ads}^{o}$ ≤ −40 kJ/mol implies there are electrons sharing or transfer from OGCI molecules to the examined metal surface to enhance formation of coordinate type of bond. This process is called chemisorption.	[175]
	For −20 kJ/mol ≤ $\Delta G_{ads}^{o}$ ≤ −40 kJ/mol, the adsorption process is a mixture between physical and chemical adsorptions.	[176]
$\Delta H_{ads}^{o}$	Positive value for $\Delta H_{ads}^{o}$ implies adsorption process is endothermic in nature while negative value suggests exothermic adsorption exhibition for tested OGCI.	[177]
$\Delta S_{ads}^{o}$	Positive value for $\Delta S_{ads}^{o}$ indicates adsorption process of OGCI on corroded metal surface under investigation in a corrosive medium is supported by an increase in entropy and vice versa.	[178]
$E_{a}$	An $E_{a}$ value of greater than 20 kJ mol^−1^ suggests inhibition process to be a controlled surface reaction.	[179]
	An increase in the value of *E*_*a*_ in the presence of OGCI indicates OGCI adsorption on examined metal surface by increasing energy barrier for corrosion process without changing the mechanism of dissolution. Also, physical adsorption (electrostatic) has occurred at the initial stage of the process.	[92, 179]
	A decrease in *E*_*a*_ at higher OGCI efficiency exhibits shift in net corrosion reaction from uncovered metal surface to adsorbed sites.	[180]
$\Delta S_{a}$	Increase in the value of Δ*S*_*a*_ in the presence of OGCI suggests increase in the degree of disorderliness resulting from conversion of reactants to activated complexes. Such exhibition could also be attributed to reduction in the release of H^+^ on metal surface making the system to shift from a more organized into a more random order thereby increasing the entropy of activation.	[181]
	If Δ*S*_*a*_ has a positive value, adsorption process is enhanced by increase in entropy which acts as a driving force for adsorption of OGCI on metal surface.	[91]
	Negative value for Δ*S*_*a*_ suggests occurrence of degree of disorderliness reduction taking place on moving from reactants to the activated states.	[182]
$\Delta H_{a}$	A positive value for *ΔH*_*a*_ implies adsorption process of OGCI on metal surface is endothermic while a negative value means exothermic reaction.	[183]
	An increase in the value of *ΔH*_*a*_ in the presence of OGCI suggests the presence of energy barrier for reaction due adsorption of OGCI.	[184]

## 3 Conclusions

### 3.1 Conclusions

The following few points were concluded after comprehensive scrutiny of literatures on this subject matter:

1. Many of the greeners considered for extraction of OGCIs are edible and are very useful for human need in many areas such as medicinal, pharmaceutical, food consumption and so on thus, making them to be very competitive in terms of functionality.
2. It has been established that many silicates also possess corrosion inhibitory attributes due to their capability to block corrosion active sites on metals in acidic medium.
3. *None* of the examined studies presented chain reaction mechanisms and reaction pathways showing present of intermediates in the course of corrosion reaction inhibitory effects of used OGCIs and examined metals.
4. Also, there is complexity of adequate separation techniques to be employed in obtaining tested acidic or alkaline solution (*as the case may be*) in pure form and the spent OGCIs.
5. The gap between research and industrial application of these OGCIs has not been bridged. From investigation, many industries are still using corrosion inhibitors already in existence which had been proved to be expensive and environmental unfriendly despite the fact that researchers are working assiduously to generate cheap, environmental friendly and readily available OGCIs from greeners. Researchers' efforts should be appreciated in this regard.
6. In the examined functional groups presented in Table 2, flavonoid was observed to be peculiar active corrosion inhibition constituent present in almost 85% of greeners presented.
7. Though synthetic inhibitors have been shown to be expensive and toxic with restrictive environmental regulations in many countries, they have high corrosion inhibition effectiveness. However, OGCIs from plants extracts with biodedradability, non-toxic and environmental friendly potential exhibit low corrosion inhibition efficiency as presented in many studies [34].
8. Many studies suggested Langmuir as the best predictive adsorption isotherm model that conforms to experimental data for corrosion inhibition of many materials by most OGCIs.
9. It has been established that organic compounds structures of OGCIs play vital role on how they effectively inhibit corrosion of metal. This means changing organic compounds chemical structure will directly sectionize corrosion inhibition.
10. Corrosion inhibition effects of OGCIs on mild steel have been the major consideration in numerous previous studies because of its relatively low price with acceptable material properties for many domestic and industrial applications [101]. However, its low corrosion resistance in acidic environments is a major challenge [136].
11. Building collapse has been linked majorly to weakening of iron steel rods (used in concrete beams) as a result of corrosion over period of time.
12. Some corrosion inhibitors extracted from *ginkgo biloba* leave [56] and *Diospyros* Kaki L.f husk [59] that have exhibited potential for corrosion inhibition for microbial induced corrosion (MIC) type need be further investigated for other forms of corrosion to widen the scope of corrosion control in oil and gas industries.
13. Acid solutions have found industrial application in mill scales removal from metallic surfaces, acid descaling, acid pickling, industrial acid cleaning and oil well acidizing. An important stimulation technique for enhancing oil production is petroleum oil well acidization. However, studies of metal corrosion using organic acid solutions were rare as compared to similar studies with mineral acids as corrosive media [185]. Among acid solutions, HCl and H_2_SO_4_ were the most widely used because of their high corrosive nature to most metals and alloys even at low concentrations [186] while HNO_3_ and H_3_PO_4_ were explored in isolated cases [187]. It is very important to put these mineral acids and organic acids into consideration during corrosion inhibition of metals using OGCIs as they were among the highly ranked important industrial chemicals.
14. Previous researchers have been using days and months to critically figure out adsorption corrosion process of examined materials, media (either acidic or alkaline) and inhibitors from greeners to truly determine percent inhibition efficiency of inhibitors. Though very few researchers have been working on methodologies and technologies to employ to reduce corrosion examination period as presented in Table 14 below, there is still need for thorough research works by many researchers on novel methodologies that can examine hundreds or thousands of materials within few minutes with optimum accuracy of inhibition efficiency determination. The most common methodologies that had been employed and adopted were EIS, WL and PDP.

**Table 14:** Employed methodologies for corrosion examination time reduction.

Materials	Methodologies	No of inhibitors	Observation period	Observation(s)	References
Aluminium alloy (AA2024)	Direct current polarization	50	9 hours	Results obtained correlated perfectly with those having over 10 days extended testing period.	[188]
Al^3+^	Fluorometric detection	14	1–7 days	Excellent results obtained with high accuracy within limited period.	[189]
Al^3+^	Direct current polarization, cyclic voltammetry and fluorometric detection	100	3–5 days	Better results with high accuracy within short period.	[190]
Fe and Zn	Scanning vibrating electrode technique	4	≈2 hrs	Accurate determination of percent corrosion inhibition efficiencies.	[191]
Carbon steel	High-throughput testing rig	88	≈1 day	Best ever methodology that can handle many inbitors within short period on a single plate with negative and positive controls.	[192]
Mild steel	High-throughput electrochemical impedance spectroscopy	12	≈3 hrs	An electrochemical platform having spatially addressable feature interfaced to a commercial EIS instrument was developed.	[193]
Mild steel	Robust computerized optical image processing method	25	≈2 hrs	A linear relationship binding image apparent grey scale value with corrosive pitting depth in the specimens was revealed.	[194]
15. Recently, researchers have written separate comprehensive review papers on plant extracts [195], ionic liquids [196], drugs [197] and amino acids [23] as major sources of OGCIs. This review paper, however, *uniquely* bridged the gap among the stated sources of OGCIs by providing useful information for prospective researchers in the field of corrosion engineering. Also, many researchers *rarely* present various isotherm and thermodynamic models used in validating their corrosion experimental data. Only results are usually presented and discussed. All applicable isotherm and thermodynamics equations that *any* researcher in this field could think of are well-presented for future usage. Nevertheless, various kinetics modelling corrosion equations for various corrosion types also make this review paper to be *superb.* Prospective researchers are implored to apply them in forcasting corrosion types and corrosion rate of materials in order to cut wasteful corrosion costs.

### 3.2 Recommendations

Current researchers whose research works center on this subject matter and prospective researchers who may develop interest after indepth understanding of this article could develop and execute novel research works in Corrosion Engineering field by considering the following stated recommendations:

1. There is need for future research works to focus more on wastes from greeners constituting environmental nuisance causing harmful effects on both human and aquatic natures. Recently, Al-Zubaidi et al. [79] used crude glycerol (byproduct of biodiesel production) with concentrations range of 0.1%–1.0% w/w as a potential OGCI for steel corrosion in a corrosive medium containing 0.5M hydrochloric acid at a constant room temperature of 25 °C.
2. Researchers should focus on synthesizing composite OGCIs from greeners extracts and silicates (such as rice husk waste) to improve their efficiency. This phenomenon is refered to as synergism effect which can be quantified by applying Eq. (7) earlier presented. This will also enable researchers to widen their scope and knowledge of using naturally endowed greeners.
3. Prospective researchers should show how intermediates produced during corrosion reaction inhibitory effects of OGCIs and metals contribute or affect the inhibition via adsorption of OGCIs on metals. It is an established fact that when extracts from greeners react, intermediates are formed in the course.
4. The reusability of spent OGCIs and decrease in their efficiency with time is a great challenge for prospective researchers in the field of Corrosion Engineering.
5. Cost effective modern techniques that will maximize OGCIs extraction in pure form from their sources are required. Detailed financial implication from pilot scale to full industrial plant is needed to expose general public to see this as a key means of internal revenue generation. Previous studies focussed majorly on refluxing greeners in HCl, ethanol and H_2_SO_4_ solutions for some period of time. In support of these, existing optimization tools such as response surface methodology and central composite design of design expert coupled with predictive tools such artificial neural network based Monte Carlo simulation, sum of square errors and so on will be of help [198].
6. Flavonoid is a good candidate to explain the corrosion inhibition effects observed in greeners. Its detailed chemistry must be studied to enhance further works on contribution of flavonoid in OGCIs on corrosion inhibition of metals.
7. Phytochemical analysis provides information on active ingredients present in plants extracts acting as corrosion inhibition of OGCIs. Therefore, it is most likely that a mixture of constituents are acting as corrosion inhibitors [20]. Few studies were engaged in using this analysis.
8. Additional advanced characterization techniques coupled with fundamental studies are required to further differentiate OGCIs mechanism and investigate relationship binding their structure with experienced corrosion inhibition. This knowledge will help tailoring OGCIs structure in obtaining necessary corrosion inhibition properties [199].
9. Further research works are required on using additives such as iron control agents, water wetting agents, anti-sludge agents, non-emulsifiers, stabilizers and viscoelastic surfactants with the green corrosion inhibitors. They do not have corrosion inhibition potential but can enhance corrosion inhibition performance of OGCIs and significantly reduce corrosion rate.
10. Deep studies are required on kinetics of corrosion inhibition of materials by OGCIs via earlier presented modelling equations. Also, more studies are needed in the aspect of developing mathematical models with reduced assumptions involving kinetics and mechanistic studies in predicting corrosion inhibitory effects of OGCIs from plant extracts. Computer software that will enhance quick prediction and other applications pertaining to corrosion inhibitory exhibits of these OGCIs can be developed.
11. Novel research works on quantitative models development to bridge chemical structure to properties using existing machine learning or statistical approaches are required. Though quantitative structure-activity relationships (QSPR) and quantitative structure-property relationships (QSPR) modelling had been presented [200, 201, 202], there is need for more machine learning modelling methods and computational models that are applicable in studying OGCIs organic compounds corrosion inhibitory properties. Also, improvements in robotics and machine learning will pave ways to tremendous increase in the efficiencies and dependency of methods for designing OGCIs within short period.
12. There is need to work assiduously on other metallic components such as copper, alloys, aluminium, stainless steel and so on. All these work in unison and play specific roles in material selection for domestic/industrial purposes. They also corrode when subjected to certain environmental conditions. They are also major components of automobiles whose corrosion is of major concern.
13. Thorough research is required on the use of OGCIs from greeners that have strong affinity for concrete cements and constituents (pastes) to increase the life span of reinforced concrete structures damaged as a result of high alkalinity and tackle rusting of these iron rods used in building construction.
14. More researches should be carried out on other corrosive environments like CO_2_, H_2_S and NaCl besides MIC type having great impact on metals used in oil and gas industries. Nevertheless, identification of corrosion type inhibited by OGCIs based on the solution examined for the materials is necessary.

## Declarations

### Author contribution statement

All authors listed have significantly contributed to the development and the writing of this article.

### Funding statement

This research did not receive any specific grant from funding agencies in the public, commercial, or not-for-profit sectors.

### Competing interest statement

The authors declare no conflict of interest.

### Additional information

No additional information is available for this paper.
