# Supplementary material for: Progress on pharmaceutical drugs, plant extracts and ionic liquids as corrosion inhibitors
Source: Heliyon. 2019 Feb 1;5(2):e01143. doi: 10.1016/j.heliyon.2019.e01143 (PMC6360517; doi:10.1016/j.heliyon.2019.e01143)
Supplement: Supplementary Table [file mmc1.docx]

**Supplementary Table 1:** Plant Extracts as OGCIs

| **OGCIs Source** | **Extraction**  **Methodology** | **MaterialTested/**  **Solution Used** | **OGCIs Characterization/ Lab. Analysis** | **Findings** | **Future Challenges** | **References** |
| --- | --- | --- | --- | --- | --- | --- |
|  |  |  |  |  |  |  |
| *Camellia sinensis* (Green Tea) | Dried and grinded leaves subjected to reflux in 70% acetone for 4 hours | Mild steel in 1M HCl | SEM, EIS, WL, FTIR, EDX, | 1. 79% inhibition efficiency achieved in 200 ppm solution.  2. Inhibition effect increase with increase in solution concentration and temperature. | 1. Adsorption kinetics and isotherms studies were not examined. | [1] |
| *Rothmannia longiflora* extract | Extraction | Mild steel in 1 M HCl and 0.5 M H_2_SO_4_ | PDP, EIS | 1. Increased in corrosion inhibition efficiency as extract concentration and temperature increase. | 1. Extraction methodology was not presented.  2. Extract was not characterized for functional groups inhibiting corrosion. | [2] |
| *Anthocleista Djalonesis* Leaf Extract | 20 g of dried leaves under reflux for 3 h in 1M HCl and 0.5M H_2_SO_4_ solutions. | Mild steel in 1 M HCl and 0.5 M H_2_SO_4_ solutions. | EIS, PDP, DFT based QCC, | 1. Corrosion inhibition via mixed-inhibition mechanism.  2. Djalonenoside (DJN) and its hydrolysis product DJN-hyd were extracts enhancing corrosion inhibition in the medium. | 1. Corrosion of other metals besides mild steel was not investigated. | [3] |
| *Theobroma cacao* peel polar extract | Boiling dried pods under reflux for 4 hrs in 1.0 M HCl. | mild steel in 1M HCl  medium | WL, EIS, PDP | 1. Increase in corrosion inhibition efficiency as OGCI concentration increases but decreased temperature rises.  2. Langmuir isotherm was obeyed. | 1. More metallic materials should be tested. | [4] |
| *Diospyros* Kaki L.f husk extracts | Husk powder heated under reflux with water or alcohol for 4 h. | Q235A steel in 1M HCl | PDP, GM | 1. Extracts behaved like a mixed type inhibitor.  2. Extracts exhibited antibacterial activity against microbial influenced corrosion (MIC) of oil field microorganism | 1. There is need to fully explore corrosion inhibitory feature of extracts from this biomass in other corrosion types besides microbial influenced corrosion.  2. SEM analysis was not carried out to ascertain that corrosion type inhibited on metal surface by the extracts was exactly MIC. | [5] |
| Schiff bases | 8 hr refluxing of 3-amino-2-methylquinazolin-4(3*H*)-one with 4-hydroxybenzaldehyde and  *N*,*N*-dimethyl-4-aminobenzaldehyde in acetic acid. | Mild steel  in 1.0 M HCl solution | SEM, NMR, DFT | 1. *para* position substituent enhanced inhibition efficiency.  2. Inhibition efficiency relies on OGCI nitrogen amount, their molecular weight and concentration. | 1. *meta* position substituent on OGCI molecule affected inhibition efficiency negatively.  2. Corrosion type prevented was not specified.  3. Only mild steel was used to test the inhibitor efficiency. | [6] |
| *Ginkgo biloba* leave extracts | Powder was  Heated with water or alcohol for 4 hrs after being dried under 60^o^C. | Q235A steel in 1M HCl Solution | PDP | 1. Extracts exhibited mixed type inhibitor behaviour.  2. Extract revealed antibacterial activity against oil field microorganism. | 1. More laboratory analysis is required to corroborate the findings.  2. Though corrosion mechanism and antibacterial activity of the extracts were presented, adsorption and kinetic studies of the process are needed in future studies. | [7] |
| *Musa sapientum* | - | mild steel in 0.5M H_2_SO_4_ | ECM | 1. Decrease in corrosion rate as inhibitor concentration increases.  2. Physisorption was experienced. | 1. Only acidic medium was examined.  2. Reaction kinetics was not studied.  3. Limited laboratory analysis. | [8] |
| Sida Acuta *(Malvaceae)* leaves | Immersion of pounded leaves into HCl solution at 15g per litre, 30g per litre and 45g per litre of 0.7M, 1.2M and 2.2M HCl. | Mild steel in 0.7M, 1.2M and 2.2M HCl | WL | 1. 71.16% maximum inhibition efficiency attained at 15g per litre of 0.7M HCl with corrosion rate reduction from 1.0485 to 0.3006 mgcm^-2^h^-1^.  2. Phytochemical analysis revealed presence of alkanoid, tannin, saponin, phytate, flavonoid and phenol.  3. Out of examined isotherms, experimental data fiited well into Langmuir. | 1. SEM image revealed degradation of mild steel surface in an uninhibited 0.7M HCl solution to be localized.  2. Only weight loss as a means of checking inhibitor efficiency was used.  3. Only acidic medium.  4. Only mild steel. | [9] |
| *Petersianthus macrocarpus* plant | Boiling dried leaves under reflux for 3 hrs in 1.0 M HCl and 0.5 M H_2_SO_4_ solutions | Mild steel in 1M HCl and 0.5M  H_2_SO_4_ solutions | GM, PDP, EIS | 1. EIS data revealed organic matter extract influence on corrosion inhibition effect on mild steel.  2. Inhibition efficiency increased with increase in concentration and temperature up to 50^o^C.  3. Lower activation energy in the presence of corrosion inhibitor resulted from adsorption chemisorptive nature. | 1. Inhibitor’s influence in alkaline medium was not investigated.  2. Only mild steel was examined.  3. Kinetics of the process was not studied.  4. Efficiency of inhibitor used was not compared with those of previous inhibitors used by researchers. | [2] |
| *Hibiscus rosa-sinensis* leaves extract | - | Mild steel in 1M HCl medium | WL, EIS | 1. Inhibition efficiency increased as temperature and solution concentration increased.  2. OGCI behaved as mixed-type.  3. Spontaneous reaction.  4. Data agreed well with Langmuir, Flory-Huggins and Freundlich adsorption isotherms. | 1. Limited laboratory analysis to affirm inhibitor’s efficiency.  2. Only mild steel in only acidic medium was investigated. | [10] |
| D-glucose derivatives | Multicomponent Reactions | Mild steel in 1M HCl | SEM, EDX, AFM, EIS | 1. Presence of -OH and -OCH_3_ groups exhibited higher inhibition efficiency. | 1. *E*_ads_ values did not exhibit any regular trend for aqueous and protonated inhibitor molecules. | [11] |
| Silica extract from rice husk ash + Na_2_O | Silica extract was prepared by mixing 80 ml 2.5 M NaOH with rice husk ash produced by calcination at 600^o^C for 6hrs. 0.2 M NaOH and distilled water were then added to form the inhibitor. | 99.9% Cu, Al alloy (AA6061), carbon steel (SAE1045) in 0.5 M HCl | XRF, XRD | 1. Each metal alloy influenced optimal SiO_2_:Na_2_O ratio determination sodium silicate formulation.  2. Used Silicate based inhibitor has a potential of inhibiting corrosion in tested samples under examined acidic medium. | 1. Limited laboratory analysis for more confirmation of inhibitor efficiency.  2. Only acidic medium solution was tested.  3. More metallic samples should be examined. | [12] |
| Gum arabic | - | Mild steel and aluminium in H_2_SO_4_ solution | WL, TT | 1. Inhibition efficiency increases with increase in concentration of inhibitor.  2. Inhibitor obeyed Temkin adsorption isotherm for tested samples.  3. Mild steel corrosion was chemical adsorption while aluminium corrosion was physical adsorption.  4. Inhibitor acted better on Al than mild steel with adsorption being spontaneous. | 1. Methodology of inhibitor extraction was not adequately presented.  2. Kinetic of adsorption process was not presented.  3. Reaction mechanism of the inhibitor adsorption process on mild steel and Al process was not available.  4. Limited laboratory analysis to support inhibitor’s efficiency on samples. | [13] |
| Coconut coir dust extract | Hydrogen evolution extraction method | Al corrosion in 1M HCl medium | WL, HEM | 1. As temperature and concentration was increased, inhibition efficiency  Increased.  2. Langmuir isotherm was obeyed. | 1. Only Al was considered. It would be better if inhibitor’s efficiency is tested in other metals.  2. Also, only HCl as acidic medium was tested. Both acidic and alkaline solutions should be checked. | [14] |
| Jatropha Stem | Jatropha fine powder obtained by sun drying and grinding soaked in ethanol for 24hrs. Evaporation of filtrate to remove excess alcohol. | Mild steel in seawater | SEM, WL, FTIR | 1. Coupons without inhibitor corroded more in seawater than those with inhibitor.  2. Presence of active corrosion inhibitors Jatropha extracts revealed by FTIR.  3. Maximum inhibition efficiency of 81.7% at 0.90g/l inhibition concentration. | 1. Adsorption isotherm and thermodynamics not studied.  2. Few laboratory analysis for more affirmation of inhibitor’s efficiency.  3. Inhibitors influence and efficiency in acidic and alkaline medium not investigated. | [15] |
| Tobacco extract | Extraction by weighing aqueous solutions, boiling off water and weighing residue. | 1008/1010 cold-rolled steel and 3105 H24 aluminum Q-panels in 1-3% NaCl solution | ZRA, PDP, WL | 1. Tobacco extracts proved to be excellent inhibitor for corrosion of aluminum and steel in alkaline solution.  2. Extract also worked in acidic solution and could prevent corrosion during descaling processes.  3. Inhibition effect greater than chromates within solution concentrations range, as low as 100 ppm. | 1. Thermodynamics, kinetics and adsorption isotherm equilibrium of inhibitor’s effect not investigated.  2. Inhibition effect in other media was not investigated. | [16] |
| *Citrus*  *aurantiifolia* Leaves | Dried and grounded leaves under reflux for 3 hrs in 1M H_2_SO_4_ solution | Mild steel in 1M HCl | SEM, WL | 1. Corrosion inhibition increases with increase in solution concentration with 97% efficiency.  2. Experimental data conformed with Langmuir isotherm | 1. Only mind steel and acidic medium investigated. | [17] |
| Cashew waste | Sun dried and pulverised fruits soaked in 250 ml of ethanol for 24 hours | Mild steel in 1M HCl and 0.1M H_2_SO_4_. | WL, SEM, FTIR | 1. Inhibitor efficiency increased with increase in inhibitor’s concentration with optimum 80.5%.  2. Cashew waste was seen as valuable corrosion inhibitor. | 1. Adsorption kinetic, isotherms and thermodynamics were not studied for indepth investigation.  2. Only mind steel and acidic medium investigated. | [18] |
| Locust bean gum | - | Carbon steel 39, 44 and B500 in H_2_SO_4_ solution | PDP, EIS | 1. Inhibition effect on Steel 39 in acidic  medium in the presence of NaCl was revealed. | 1. Though different carbon steel samples were tested, there was shallow investigation of the extracted corrosion inhibitor on the examined samples. | [19] |
| Banana peel extract + Zn | - | carbon steel in distilled water | AFM, WL, GM, FTIR | 1. Zn addition decreased inhibition efficiency. It later increases after increasing Zn concentration. | 1. No mathematical model was presented as predictive tool for future corrosion of sample tested. | [20] |
| Fig leaves extract | Extraction by reflux in soxhlet extractor for 5hrs in alcohol | Mild steel in 2M HCl | WL, EIS | 1. Mixed type inhibitor formed.  2. Data support Langmuir isotherm.  3. 87% inhibition efficiency with 200ppm of inhibitor’s solution. | 1. Only mild steel in acidic medium examined. | [21] |
| Rice straw extract | Reflux of 10 g of rice straw in 100 mL of 1M HCl for 1 hour | Mild steel in 1M HCl | EIS, PDP | 1. Physisorption of rice straw extract on mild steel electrode surface.  2. Both anodic and cathodic reactions were inhibited while OGCI exhibited mixed type.  3. Adsorption data obeyed langmuir isotherm. | 1. Few characterization of inhibitor extracted.  2. Only mild steel in hydrochloric acidic medium alone. | [22] |
| *Dialum guineense* and *Euphorbia hirta* Leaf extracts | Some grams of dried leaves subjected to reflux for some hours 0.5M H_2_SO_4_ solution. | Al alloy (AA8011) in HCl solution | GM | 1. Both extracts behaved as good inhibitors.  2. Inhibition efficiency improved with concentration.  3. Experimental data conformed with langmuir adsorption  Isotherm. | 1. Limited laboratory analysis for more investigation of inhibitors’ efficiencies. | [23] |
| Extract of *Ficus asperifolia* | Pulverized stem was obtained after oven drying at 40^o^C. Powdered stem bark was then soaked in 80% ethanol for 72 hours. | Mild steel in 5M HCl acid solution | WL, SEM, FTIR, AAS | 1. Inhibition efficiency increased with increase in extracts concentration but decreased with temperature.  2. Inhibition of corrosion resulting from mixed plant constituents adsorption on surface of metal by spontaneous reaction.  3. Extracts contain tannins, alkaloids, anthraquinones, flavonoids, saponins and reducing sugars.  4. Kinetic studies revealed data agreed with first order reaction. | 1. Though detailed kinetic, isotherm and thermodynamics studies of adsorption process was presented, limited laboratory analysis for inhibitor’s characterization was done.  2. Only mild steel in acidic medium was examined. | [24] |
| Seeds of *Areca*  *catechu* | After drying and grinding, seeds were subjected to reflux for 8hours in ethanol solution. | Mild steel in 0.5M HCl | WL, PDP, EIS, SEM, FTIR | 1. Mixed type inhibitor revealed.  2. 96.97% corrosion inhibition achieved at 500 ppm of inhibitor’s concentration.  3. Experimental data agreed with Temkin isotherm. | 1. Only mild steel examined.  2. Higher inhibitor concentration required to achieved better corrosion inhibition. | [25] |
| Elephant grass extract (Pennisetum purpureum) | 10g of dried and pulverized leaves soaked in 100ml of ethanol for 48 h. Filtrates further subjected to evaporation to obtain ethanol-free sample. | Mild steel in 1M HCl medium | AAS, SEM, FTIR, | 1. Steel dissolution rate sensitive to extract concentration in acidic solution. Mass loss and corrosion rates decreased with increase in extract concentration.  2. Inhibition influenced by absorption via presence of extract functional groups.  3. Langmuir adsorption isotherms and activation energies revealed physical adsorption.  4. SEM images of corroded substrates showed primary corrosion mechanism to be by pitting.  5. 95% Inhibition efficiencies at room temperature achievable.  6. Corrosion inhibition increased with increase in extract concentration but decreased with increasing temperature. | 1. Though detailed work was done, inhibition effectiveness of elephant grass extract was only tested using mild steel in HCl as acidic medium. | [26] |
| Guar gum | 1. Pods dried in sunlight and  Separated manually from seeds.  2. Seeds heated under reflux with water or alcohol for 6 hrs. | carbon steel in 1M  H_2_SO_4_ solution + NaCl | WL, EIS, PDP | 1. Increase in resistance of pitting corrosion was exhibited.  2. Guar gum acted as a mixed-type inhibitor whose efficiency increases with increase in concentration.  3. All data supported Langmuir adsorption  isotherm. | - | [27] |
| Oil palm frond | Nitrobenzene oxidation method for lignin depolymerization | Mild steel in 1M HCl medium | WL, PDP, EIS, SEM, XRD | 1. Inhibition efficiency increased with increased concentrations lignin depolymerized products.  2. Mixed type inhibitors revealed.  3. Experimental data well fitted with Langmuir adsorption isotherm.  4. Adsorption was dominated by physisorption.  5. SEM revealed reduction of surface roughness in the presence of inhibitor. | 1. Only Langmuir isotherm was used. For comparative purposes, other existing isotherms should be used.  2. Oil palm frond extracts has been shown to have potential of corrosion inhibition in alkaline medium. Thus, various types of metallic materials should checked in alkaline medium. | [28] |
| Celery seeds (*Apium graveolens*) | Grounded and powdered seed boiled in distilled H_2_O for 2 hrs. Filtrate evaporated to dryness and residue used high concentrated stock solution | Carbon steel in 1M HCl | WL, PDP | 1. Optimum inhibition efficiency obtained at 500 ppm of inhibitor’s concentration.  2. Spontaneous adsorption process that conforms with Temkin isotherm.  3. Percent inhibition efficiency decreased with increased temperature.  4. Inhibition efficiency increased as Celery doses increased. | 1. Only weight loss and potentiodynamic polarization was used to check inhibitor’s efficiency.  2. Only HCl solution was used for carbon steel alone to check OGCI efficiency.  3. Active functional groups present in *Apium graveolens* seeds enhancing corrosion inhibition were not deeply investigated | [29] |
| *Eichhornia crassipes* (water hyacinth) leaves and roots | 4g of dried and ground leaves and roots soaked in 1000 ml of 5M HCl solution. | Mild Steel in HCl | DFT, GT | 1. Root and leaf extracts performed excellently well as effective OGCIs.  2. Physisorption of extracts organic constituents on corroding mild steel surface. | 1. Insufficient laboratory analysis.  2. Equilibrium isotherms and kinetics not investigated. | [30] |
| *Grewia venusta* plant extract | *Grewa venusta* cut into pieces, dried for 3 days and grounded into powder. Product was refluxed for some hours using ethanol. | Mild Steel in 0.5M Sulphuric Acid | SEM, ICP-OES | 1. Corrosion rate was reduced when OGCI concentration was increased above 2% v/v with time.  2. Increase in temperature massively increased corrosion rate.  3. Plant extract exhibited effective corrosion inhibition potential for mild steel in acidic medium.  4. At 8% v/v optimum concentration of plant extract in acid solution, 86.47% highest efficiency was obtained. | 1. Only SEM was used to authenticate inhibitor’s efficiency.  2. Thermodynamics of adsorption process not studied. | [31] |
| Crude Glycerol from residue of biodiesel produced from a plant seed | Transesterification process | Steel in 0.5M HCl at 25^o^C | WL, SEM, PDP | 1. Corrosion inhibition increased with inhibitor concentration.  2. Maximum inhibition efficiency of 98%) was achieved after 70hrs of residence time with 1% inhibitor concentration. | 1. Plant source of oil used for biodiesel production from which glycerol was obtained was not mentioned.  2. Inhibition efficiency remained unchanged after residence time. | [32] |

**Note:** EIS = Electrochemical Impedance Spectroscopy, AFM = Atomic Force Microscopy, SEM = Scanning Electron Microscopy, EDX = Energy Dispersive X-ray Spectroscopy, PDP = Potentiodynamic polarization, DFT = Density functional theory, QCC = Quantum chemical computation, FTIR = Fourier Transform Infra Red, NMR = Nuclear Magnetic Resonance, SRB = Sulfate reducing bacteria, IB = Iron bacteria, TGB = Total general bacteria, GM = Gravimetric method, LPR = Linear polarization resistance, TFM = Tafel extrapolation method, ST = Surface tension, XRF = X-ray fluorescence, XRD = X-ray diffraction, WL = Weight loss, TT = Thermometric techniques, ZRA = Zero-resistance ammeter, ECM = Electro-chemical measurements, HEM = Hydrogen evolution method, AAS = Atomic absorption spectroscopy, GT = Gasometric technique, ICP-OES = Inductively Coupled Plasma Optical Emission Spectroscopy.

**References**

[1] Nofrizal AI (2012) Corrosion inhibition of mild steel in 1M HCl by catechin monomers from commercial Green tea extracts, Scientific Contributions Oil & Gas, 35(1):11-24. https//www.hindawi.com/journals/ijc/2012/897430

[2] Akalezi CO, Oguzie EE, Ogukwe CE, EJele EA (2015) *Rothmannia longiflora* extract as corrosion inhibitor for mild steel in acidic media. Int J Ind Chem, 6:273–284.DOI: 10.1007/s40090-015-0050-z

[3] Ogukwe CE, Akalezi CO, Chidiebere MA, Oguzie KL, Iheabunike ZO, Oguzie EE (2012) Corrosion Inhibition and Adsorption of *Anthocleista Djalonesis* Leaf Extract on the Acid Corrosion of Mild Steel. *Portugaliae Electrochimica Acta*, 30(3):189- 200. DOI:10.4152/pea.201203189

[4] Yetri Y, Jamarun E, Gunawarman N (2014) Corrosion inhibition efficiency of mild steel in hydrochloric acid by adding Theobroma cacao peel extract. International conference on biological, chemical and environmental sciences, Penang (Malaysia).

[5] Zhang J, Song Y, Su H, Zhang L, Chen G, Zhao J (2013) Investigation of *Diospyros* Kaki L.f husk extracts as corrosion inhibitors and bactericide in oil field. Chemistry Central Journal, 7(1):109. DOI: 10.1186/1752-153X-7-109

[6] Jamil DM, Al-Okbi AK, Al‑Baghdadi SB, Al-Amiery AA, Kadhim A, Gaaz TS, Kadhum ARH, Mohamad AB (2018) Experimental and theoretical studies of Schiff bases as corrosion inhibitors. Chemistry Central Journal, 12:7. DOI: 10.1186/s13065- 018-0376-7

[7] Chen G, Zhang M, Zhao JR, Zhou R, Meng ZC, Zhang J (2013) Investigation of Ginkgo biloba leave extracts as corrosion and oil field microorganism inhibitors. Chem Central J 2013, 7:83. DOI: 10.1186/1752-153X-7-83

[8] Salami L, Wewe TOY, Akinyemi OP, Patinvoh RJ (2012) A Study of the Corrosion Inhibitor of Mild Steel in Sulphuric Acid using *Musa Sapientum* Peels Extract. Global Engineers & Technologists Review, 2(12).

[9] Ndukwe AI, Anyakwo CN (2017) Modelling of Corrosion Inhibition of Mild Steel in Hydrochloric Acid by Crushed Leaves of Sida Acuta (Malvaceae). International Journal of Engineering and Science, 6(1):22-33. DOI: 10.9790/1813-0601032233

[10] Desai PS (2015) Hibiscus rosa-sinensis (Jasud) leaves extracts used as corrosion inhibitors for mild steel in hydrochloric acid. E-jpmr 2(1):470-485. https://www.researchgate.net/publication/277570623

[11] Levy AV (2002) The erosion-corrosion behavior of protective coatings. Surface and Coatings Technology, 36(1-2):387-406. https://www.researchgate.net/publication/222738072

[12] Mohamad N, Othman NK, Jalar A (2013) Investigation of SiO_2_:Na_2_O ratio as a Corrosion Inhibitor for Metal Alloys, AIP Conference Proceedings 1571, 136. DOI: 10.1063/1.4858643

[13] Umoren SA (2008) Inhibition of aluminium and mild steel corrosion in acidic medium using gum arabic. Cellulose 15:751-761. <https://doi.org/10.1007/s10570-> 008-9226-4

[14] Umoren SA, Ebenso EE, Okafor PC, Ekpe UJ, Ogbobe O (2006) Effect of halides on the corrosion inhibition of mild steel in alkaline medium using polyvinyl alcohol. J Appl Polymer Sci. 103:2810-2816. https://doi.org/10.1002/app.25446

[15] Olawale O, Adekunle OF, Adesoji AA, Sunday OA (2016) Corrosion Inhibition of Mild Steel in Seawater using Jatropha Stem. Analele Universităţii, 23(1):228-238.

[16] Davis GD, Anthony von FJ, Krebs LA, Dacres CM (2001) The Use of Tobacco Extracts as Corrosion Inhibitors, Corrosion2001 Paper 1558.

[17] Saratha R, Priya SV, Thilagavathy P (2009) Investigation of Citrus aurantiifolia leaves extract as corrosion inhibitor for mild steel in 1M HCl, E-Journal of Chemistry, 6(3):785-795. https://www.researchgate.net/publication/242144748

[18] Olawale O, Bello JO, Akinbami P (2015) A Study on Corrosion Inhibitor of Mild- Steel in Hydrochloric Acid Using Cashew Waste. IJMER, 5(8):1-7. https://www.researchgate.net/publication/298052748

[19] Jano A, Lame A, Kokalari E (2012) Use of extracted green inhibitors as a friendly choice in corrosion protection of low alloy carbon steel. Kem Ind 61(11-12):497-503. https://hrcak.srce.hr/92460

[20] Sangeetha M, Rajendran S, Sathiyabama J, Prabhakar P (2012) Eco-friendly extract of banana peel as corrosion inhibitor for carbon steel in sea water. J Nat Prod Plant Resour, 2(5):601-610. DOI: 10.5402/2012/370802

[21] Taleb HI, Mohamed AZ (2011) Corrosion Inhibition of Mild Steel using Fig Leaves Extract in Hydrochloric Acid Solution. International Journal of Electrochemical Sciences, 6:6442-6455. http://www.electrochemsci.org/papers/vol6/6126442.pdf

[22] Mahross MH, Naggar AH, Elnasr TAS, Abdel-Hakim M (2016) Effect of Rice Straw Extract as an Environmental Waste Corrosion Inhibitor on Mild Steel in an Acidic Media. Chemistry of Advanced Materials, 1(1):6-16. https://issres.net/jbsr/pubsystem/index.php/cam/article/view/28/55

[23] Anozie IU, Akoma CS, Nnanna LA (2011) Corrosion inhibition of aluminium alloy in acidic medium by Euphorbia hirta and Dialum guineense extracts. Int J Pure Appl Sci Technol 6(2):79-88. https://www.researchgate.net/publication/268002830

[24] Fadare OO, Okoronkwo AE, Olasehinde EF (2016) Assessment of anti-corrosion potentials of extract of *Ficus asperifolia* -Miq (Moraceae) on mild steel in acidic medium. African Journal of Pure and Applied Chemistry, 10(1):8-22. DOI: 10.5897/AJPAC2015.0651

[25] Kumar KPV, Pillai MSN, Thusnavis GR (2011) “Green corrosion inhibitor from seed extract of Areca catechu for mild steel in hydrochloric acid medium”, J Mater Sci., 46:5208-5215. http://www.jmst.org/EN/article/downloadArticleFile

[26] Alaneme KK, Olusegun SJ, Alo AW (2016) Corrosion inhibitory properties of elephant grass (Pennisetum purpureum) extract: Effect on mild steel corrosion in 1M HCl solution. Alexandria Engineering Journal, 55:1069-1076. DOI: 10.1016/j.aej.2016.03.012

[27] Abdallah M (2004) Guar gum as corrosion inhibitor for carbon steel in sulphuric acid solutions. Port Electrochim Acta, 22:161-175. https://doi.org/10.4152/pea.200402161

[28] Shah AM, Abdul-Rahim A, Mohamad AMN, Hazwan MA (2017) Depolymerized Oil Palm Frond (OPF) Lignin Products as Corrosion Inhibitors for Mild Steel in 1 M HCl. *Int. J. Electrochem. Sci.,* 12:9017-9039. DOI: 10.20964/2017.10.66

[29] Megahed HE, Sobhi M, Nour S (2017) Celery (Apium graveolens) Extract as Corrosion Inhibitor for carbon steel in 1 M HCl. Journal of Basic and Environmental Sciences, 2:170-177. http://jbesci.org/published/4.2.6.pdf

[30] Ulaeto SB, Ekpe UJ, Chidiebere MA, Oguzie EE (2012) Corrosion Inhibition of Mild Steel in Hydrochloric Acid by Acid Extracts of *Eichhornia Crassipes*. International Journal of Materials and Chemistry, 2(4):158-164. DOI: 10.5923/j.ijmc.20120204.08

[31] Suleiman IY, Oloche OB, Yaro SA (2013) The Development of a Mathematical Model for the Prediction of Corrosion Rate Behaviour for Mild Steel in 0.5M Sulphuric Acid. ISRN Corrosion, Article ID 710579, 1-10. DOI:10.1155/2013/710579

[32] Al-Zubaidi I, Jones R, Al-Zughaibi M, Albayyadhi M, Darzi F, Ibrahim H (2018) Crude Glycerol as an Innovative Corrosion Inhibitor. Appl. Syst. Innov., 1(12): 1-15. DOI:10.3390/asi1020012
